# WDR5‐H3K4me3 Epigenetic Axis Promotes TRMT6‐Dependent tRNA M^1^A Modification to Facilitate Triple‐Negative Breast Cancer Progression by Suppressing Ferroptosis

**DOI:** 10.1002/advs.202513277

**Published:** 2025-12-14

**Authors:** Yuqing Lei, Xue Wang, Jiahe Liu, Zixu Niu, Jiaqi Hua, Qian Guo, Dan Du, Yuanchang Zhu, Fucheng He, Mingxia Zhou, Hongle Li, Jing He, Jun Li

**Affiliations:** ^1^ Department of Molecular Pathology The Affiliated Cancer Hospital of Zhengzhou University Zhengzhou 450008 China; ^2^ Department of Medical Laboratory The First Affiliated Hospital of Zhengzhou University Zhengzhou 450052 China; ^3^ Department of Breast Surgery The First Affiliated Hospital of Zhengzhou University Zhengzhou 450052 China; ^4^ Department of Rhinology The First Affiliated Hospital of Zhengzhou University Zhengzhou 450052 China; ^5^ Department of Gastroenterology The Fifth Affiliated Hospital of Zhengzhou University Zhengzhou 450052 China; ^6^ Department of Gastroenterology The First Affiliated Hospital of Zhengzhou University Zhengzhou 450052 China

**Keywords:** ferroptosis, N^1^‐methyladenosine, TNBC, TRMT6, WDR5

## Abstract

N^1^‐methyladenosine (m^1^A) modification widely occurs in various RNAs, yet its pathophysiological function in tumorigenesis remains poorly understood. Notably, the expression and biological roles of tRNA m^1^A methyltransferase 6 noncatalytic subunit (TRMT6) in breast cancer, particularly in triple‐negative breast cancer (TNBC), are unknown. Herein, it is demonstrated that TRMT6 is markedly elevated in TNBC, due to the H3K4me3 methylation modification occurring at the promoter region to enhance transcription. Through integrated analyses of m^1^A tRNA methylated RNA immunoprecipitation sequencing, ribosome profiling sequencing, RNA sequencing and data‐independent acquisition mass spectrometry, ferritin heavy chain 1 (FTH1) is identified as a downstream target of TRMT6. Mechanistically, it is found that TRMT6 is responsible for the formation of m^1^A methylation on tRNAs, which increases the translation efficiency of FTH1. Besides, TRMT6 promotes ferritin light chain (FTL) expression at both transcriptional and translational levels, further reinforcing its role in iron metabolism. TRMT6 regulates the malignant progression of TNBC by modulating ferroptosis in tumor cells. Conclusively, the findings indicate that histone methylation‐driven TRMT6 is crucial for the translation of FTH1 and FTL, which bridges the understanding of m^1^A tRNA modification and ferroptosis. These results highlight TRMT6 as a novel potential therapeutic target for TNBC.

## Graphical abstract

Upregulated TRMT6 forms aberrant hypermethylation of a specific tRNA pool and serves as a predictor of poor prognosis in TNBC. This m^1^A modification in tRNAs enhances translation of FTH1 and FTL, reducing the pool of bioavailable Fe^2^⁺. Reduced Fe^2+^ availability impairs RSL3‐induced lipid peroxidation and tumor progression. This study reveals a potential therapeutic strategy leveraging tRNA m^1^A dependent iron metabolism reprogramming in TNBC.

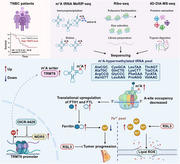

## 1 Introduction

Breast cancer (BC) is the most prevalent malignancy in women worldwide, with triple‐negative breast cancer (TNBC) representing a particularly aggressive subtype, accounting for 10–15% of all cases.^[^ ^1^ ^]^ Defined by the absence of estrogen receptor, progesterone receptor, and human epidermal growth factor receptor 2 (HER2) expression, TNBC is characterized by a high proliferation rate, early metastatic potential, and poor prognosis compared to hormone receptor‐positive or HER2‐enriched subtypes.^[^ ^2^ ^]^ Consequently, patients with TNBC often face limited treatment options and rely primarily on surgery, chemotherapy, and radiation.^[^ ^3^ ^]^ Moreover, metastatic TNBC has a median overall survival of only 12‐15 months, highlighting the urgent need for novel therapeutic targets.^[^ ^4^ ^]^

Transfer RNA serves as the foundation of protein synthesis, functioning as the bridge connecting amino acids and the nucleotide triplet genetic code.^[^ ^5^, ^6^ ^]^ In human cells, tRNA exists in tens of millions of transcripts and is the most abundant RNA in moles among all cellular RNAs.^[^ ^7^, ^8^ ^]^ The levels of tRNA abundance, modification, and aminoacylation influence mRNA decoding, reflecting cell type and its environment.^[^ ^8^, ^9^, ^10^, ^11^ ^]^ Notably, the m^1^A modification at the highly conserved position 58 of tRNA is subject to precise spatiotemporal regulation in eukaryotes.^[^ ^12^ ^]^ m^1^A methylation in tRNA is introduced via post‐transcriptional modification by tRNA m^1^A methyltransferase 6 noncatalytic subunit (TRMT6) and the catalytic subunit of tRNA methyltransferase 61A (TRMT61A).^[^ ^13^, ^14^ ^]^ Growing evidence links m^1^A methylation in tRNA to human diseases. For example, recent studies have shown that m^1^A modification contributes to colorectal tumorigenesis by triggering histone synthesis and activating cell cycle signaling.^[^ ^15^ ^]^ However, the function of tRNA m^1^A modification associated with TNBC remains poorly understood.

Here, we aimed to investigate the mechanisms underlying the role of abnormal TRMT6‐regulated tRNA m^1^A modifications in promoting TNBC progression. We found that WD Repeat‐Containing Protein 5 (WDR5) upregulates the trimethylation of lysine 4 on histone H3 (H3K4me3), which mediates the transcriptional activation of TRMT6. Abnormal TRMT6 expression mediates m^1^A modification on specific tRNAs (e.g., tRNA Ala and tRNA Asn) to enhance ferritin heavy chain 1 (FTH1) translation, thereby affecting ferroptosis in TNBC cells. Overall, we revealed the critical role of TRMT6, under the mediation of histone modifications, in inhibiting ferroptosis via tRNA m^1^A modification to expedite the malignant progression of TNBC.

## 2 Results

### 2.1 TRMT6 is Highly Expressed in TNBC Tissues and Substantially Associated with Poor Prognosis

**Figure 1:**
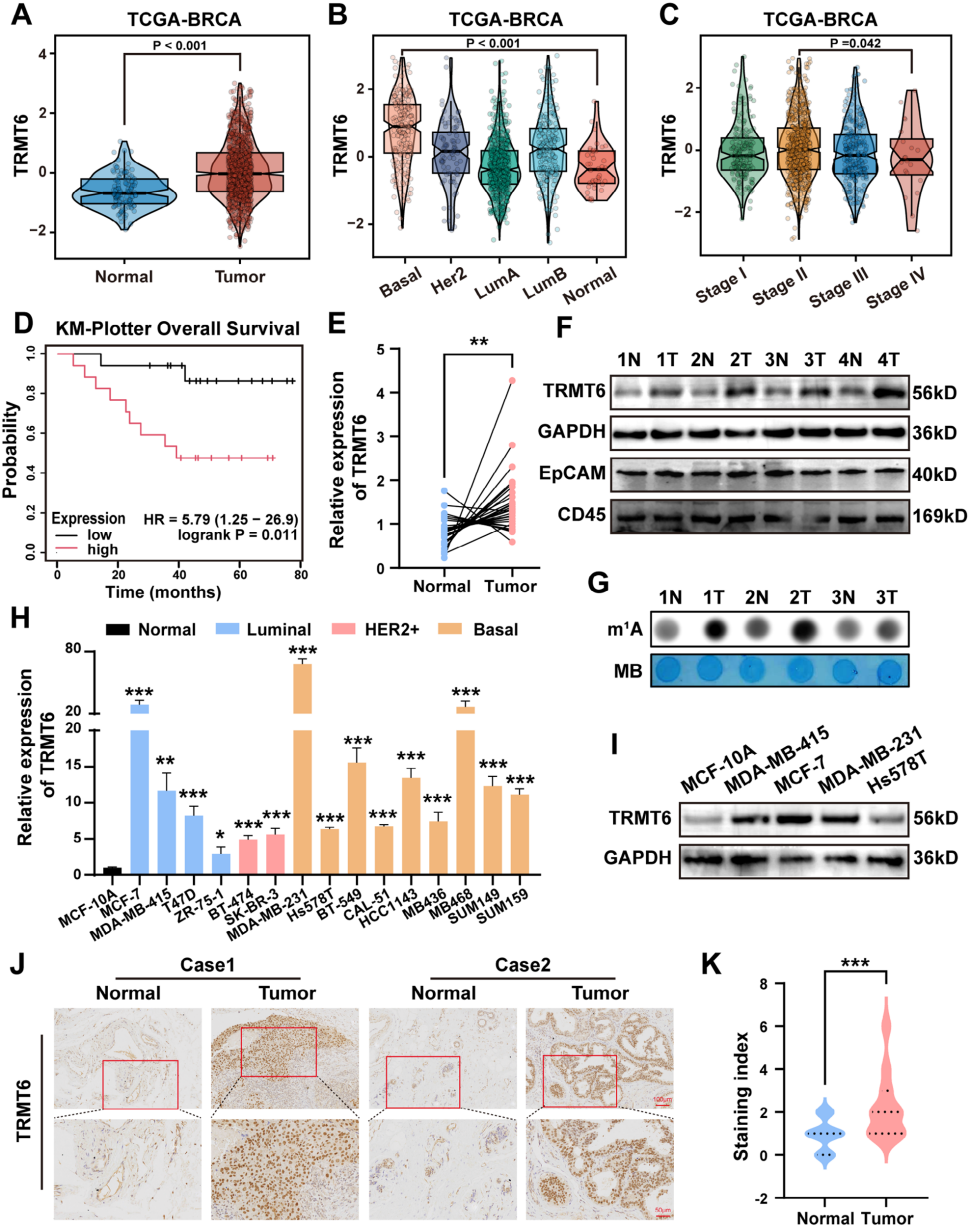
Upregulated TRMT6 expression in TNBC samples. A) The expression level of TRMT6 in normal tissues and BRCA cancer tissues from TCGA dataset. B,C) Stratification of TRMT6 mRNA expression levels in BC tissues based on different subtypes (B) and tumor stages (C). D) Correlation between TRMT6 expression and overall survival of TNBC patients according to the Kaplan–Meier Plotter database (*n* = 34). E) RT‐qPCR analysis revealed a significant increase of TRMT6 expression in TNBC tissues compared to adjacent normal tissues. F) Western blotting of TRMT6 protein levels in four pairs of human TNBC specimens. N: Normal, T: Tumor. GAPDH was used as a loading control. The expression of EpCAM (epithelial cell marker) and CD45 (pan‐immune cell marker) was assessed to account for potential variations in tissue cellular composition. G) Dot blot of tRNA m^1^A modification levels in four pairs of human TNBC specimens. H,I) RT‐qPCR (H) and Western blotting (I) of TRMT6 mRNA and protein levels in BC cell lines. J,K) Representative images (J) and quantification (K) of TRMT6 immunostaining in TNBC specimens and adjacent normal tissues. Scale bar = 50 µm/100 µm. Significant differences were shown by ^*^ *p* <0.05, ^**^ *p* <0.01, and ^***^ *p* <0.001.

To explore the mechanism of tRNA methylation modification in BC, we focused on the expression pattern and clinical correlation of TRMT6. First, pan‐cancer analysis in the TIMER database (http://timer.comp‐genomics.org/) showed that TRMT6 expression levels in BC were markedly higher than those in most other cancer types (Figure S1A, Supporting Information). Analysis of The Cancer Genome Atlas Breast Cancer (TCGA‐BRCA) dataset revealed that TRMT6 mRNA levels were significantly upregulated in BC tissues compared to adjacent normal tissues, with the most pronounced increase observed in TNBC (**Figure** **1**A,B). Besides, TRMT6 expression was robustly increased in Stage II tumors compared to Stage IV, indicating its key role in the early development of tumors (Figure 1C). Further analysis using the Kaplan–Meier Plotter database showed that the Overall Survival (OS) time of patients with high TRMT6 expression was markedly shorter than that of low‐expression patients, supporting its clinical association with TNBC malignancy (Figure 1D). Subsequent Reverse Transcription Quantitative Real‐Time Polymerase Chain Reaction (RT‐qPCR) in 27 pairs of clinically matched samples demonstrated elevated TRMT6 levels in TNBC tissues compared to adjacent normal tissues (Figure 1E). Consistently, western blotting confirmed higher TRMT6 protein expression in TNBC tissues (Figure 1F). Then dot blot analysis was performed to verify the increased m^1^A modification on tRNA in TNBC tissues (Figure 1G). Expression profile analysis of cell lines showed that TRMT6 mRNA and protein levels in TNBC cell lines were much higher than those in normal mammary epithelial cells (MCF‐10A), consistent with the higher pathologic grade of TNBC (Figure 1H,I). Additionally, immunohistochemical (IHC) staining of 27 TNBC clinical specimens demonstrated predominant nuclear localization of TRMT6 (Figure 1J). Quantitative IHC scoring further confirmed statistically higher TRMT6 protein expression in TNBC tissues than in adjacent normal tissues (Figure 1K).

### 2.2 Overexpression of TRMT6 Drives TNBC Cell Proliferation, Migration, and Cell Cycle Progression

**Figure 2:**
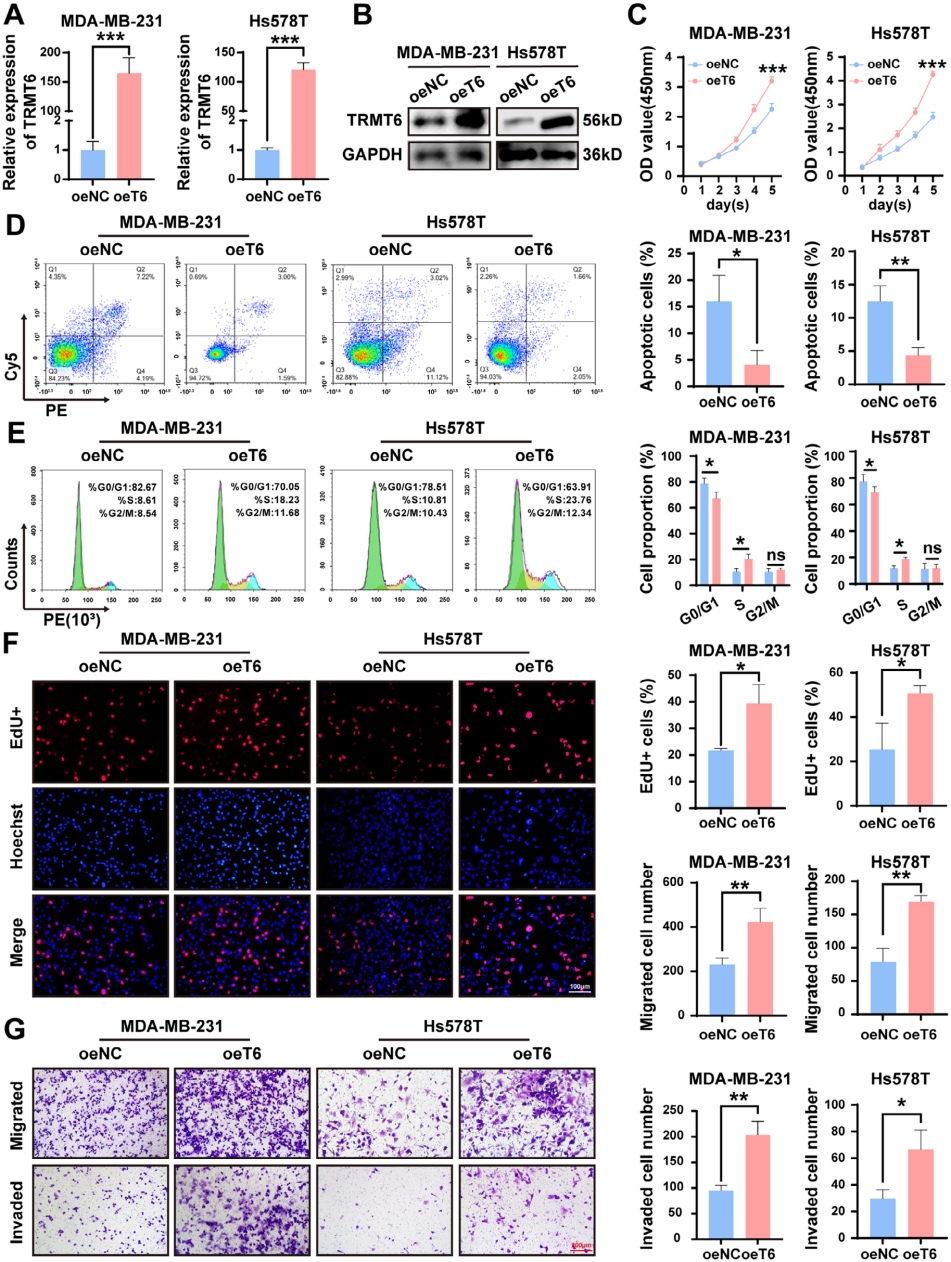
Overexpression of TRMT6 promotes TNBC progression in vitro. A) RT‐qPCR analysis of relative TRMT6 levels after over‐expression of TRMT6 and wild‐type in MDA‐MB‐231 and Hs578T cells. B) Western blotting confirmation of TRMT6 in indicated MDA‐MB‐231 and Hs578T cells. C) CCK‐8 was used to assess the effects of TRMT6 overexpression on the cell proliferation ability of MDA‐MB‐231 and Hs578T cells. D) Flow cytometric analysis of apoptotic cells percentage. E) Flow cytometric analysis of cell cycle distribution. F) EdU assays to evaluate changes in TNBC cell proliferation. G) Transwell assays to profile cell migration and invasion capacity. Scale bar = 100 µm/200 µm. Significant differences were shown by ^*^ *p* <0.05, ^**^ *p* <0.01, and ^***^ *p* <0.001.

To investigate the biological function of TRMT6 in TNBC, we performed gain function studies in TNBC cell lines. Using MDA‐MB‐231 and Hs578T cells as in vitro models, we established overexpression of the TRMT6 gene (oeT6) cell lines through lentiviral transduction (**Figure** **2**A,B). Functional assays demonstrated that overexpression of TRMT6 exhibited enhanced proliferative capacity and colony formation compared to control cells (Figure 2C; Figure S1B, Supporting Information). Flow cytometry analysis revealed that TRMT6 overexpression suppressed apoptosis and altered cell cycle distribution in TNBC cells, with a decreased proportion of cells in G0/G1 phase and an increased proportion in S phase, suggesting TRMT6 overexpression facilitates G1/S transition (Figure 2D,E). The EdU staining assays further validated the promotional effect of TRMT6 overexpression on cell proliferation (Figure 2F). Additionally, TUNEL staining confirmed the anti‐apoptotic effect of TRMT6 overexpression (Figure S1C, Supporting Information). Transwell assays showed that TRMT6 overexpression markedly accelerated cell migration and invasion (Figure 2G). Taken together, these results illustrated that TRMT6 is a key factor driving the malignant progression of TNBC cells.

### 2.3 Knockdown of TRMT6 Retards Carcinogenesis of TNBC Cells

**Figure 3:**
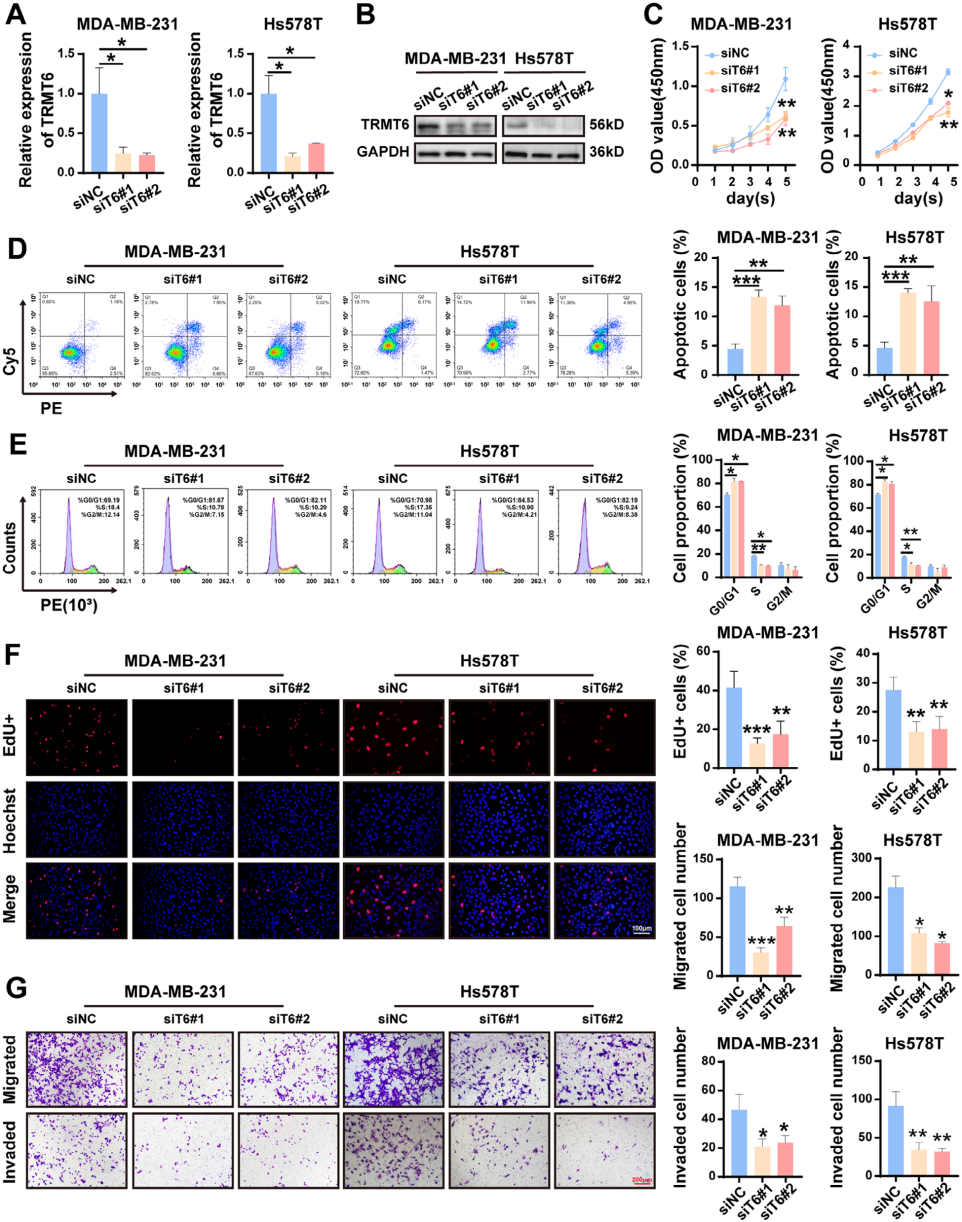
Inhibition of TRMT6 impedes BC progression in vitro. A) RT‐qPCR analysis of relative TRMT6 expression after TRMT6 knockdown in MDA‐MB‐231 and Hs578T cells. B) Verification of TRMT6 knockdown by western blotting in MDA‐MB‐231 and Hs578T cells. C) CCK‐8 was used to assess the effects of TRMT6 silence on the cell proliferation ability of MDA‐MB‐231 and Hs578T cells. D) Flow cytometric analysis of percentage of apoptotic cells. E) Flow cytometric analysis of cell cycle distribution. F) EdU assays was used to evaluate cell proliferation. G) Transwell assays was used to analyze cell migration and invasion ability. Scale bar = 100 µm/200 µm. Significant differences were shown by ^*^ *p* <0.05, ^**^ *p* <0.01, and ^***^ *p* <0.001.

To further validate the effect of TRMT6 on TNBC progression, we transfected MDA‐MB‐231 and Hs578T with two independent small interfering RNAs (siRNAs) targeting TRMT6 (si‐T6#1 and si‐T6#2). The efficient knockdown of TRMT6 mRNA and protein levels was confirmed by RT‐qPCR and western blotting (**Figure** **3**A,B). Functional experiments showed that TRMT6 knockdown considerably inhibited the proliferation and colony formation of TNBC cells (Figure 3C; Figure S1D, Supporting Information). Flow cytometry revealed that TRMT6 knockdown increased apoptosis and induced G0/G1 phase arrest, with a reduced proportion of cells in S phase (Figure 3D,E). Consistently, EdU staining confirmed cell proliferative capability was dramatically decreased following TRMT6 knockdown (Figure 3F), while TUNEL assays further validated TRMT6 knockdown promoted apoptosis in TNBC cells (Figure S1E, Supporting Information). Moreover, Transwell assays further confirmed that TRMT6 knockdown significantly suppressed the migration and invasion of TNBC cells (Figure 3G). Collectively, these results indicated that silencing of TRMT6 remarkably inhibits the malignant phenotype of TNBC.

### 2.4 WDR5 Activates TRMT6 Promoter Transcription via H3K4me3 Modification

**Figure 4:**
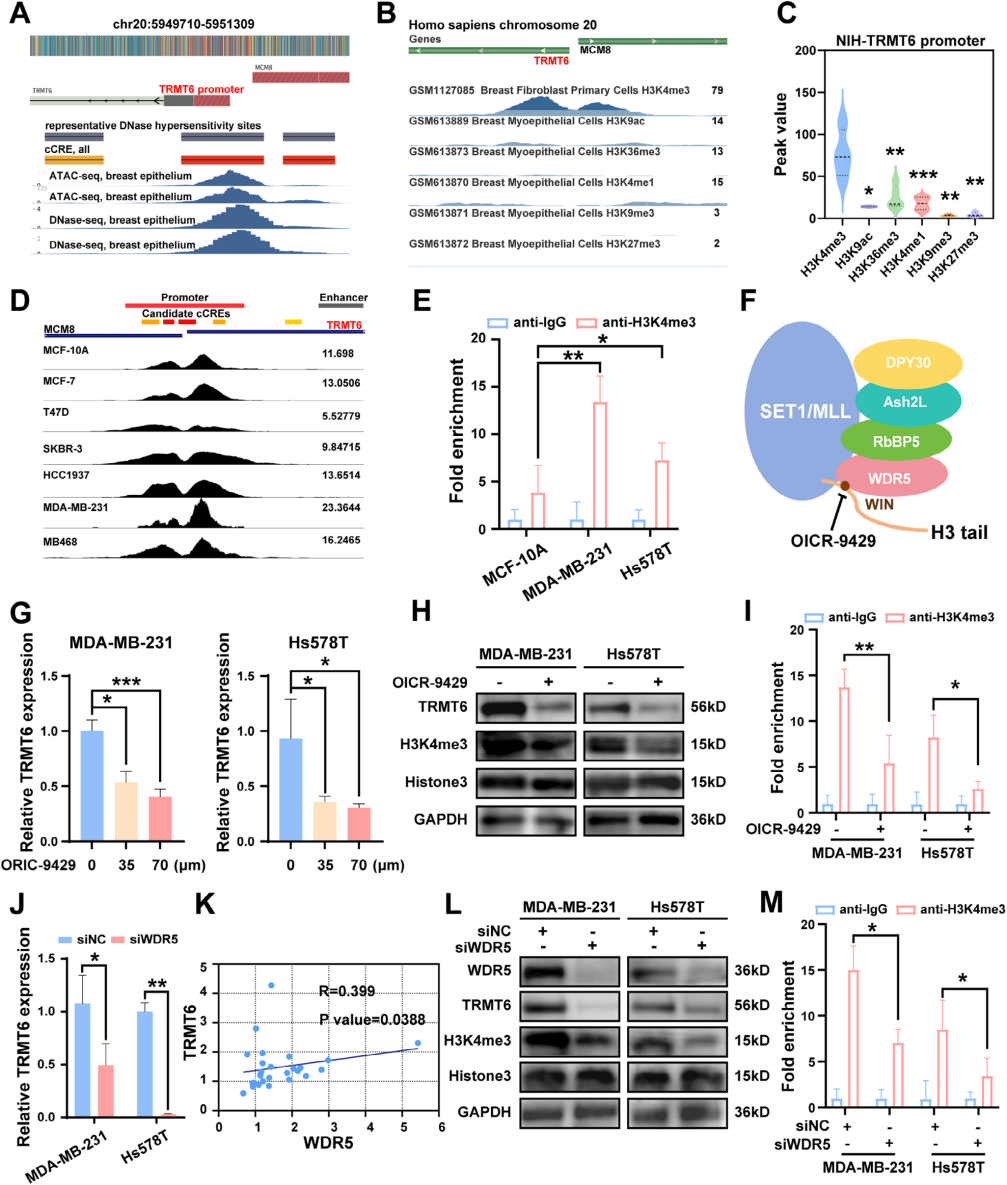
Increased WDR5 mediates the high expression of TRMT6 via H3K4me3. A) ATAC‐seq and DNase‐seq peaks, and cCREs in the TRMT6 promoter region from ENCODE. B) Histone methylation and acetylation peaks in the TRMT6 promoter region from the NIH Database. C) Statistical summary of peaks for various histone modifications from the NIH. D) The display of H3K4me3 peaks located in the TRMT6 promoter region in breast cell lines. E) ChIP‐qPCR was used to confirm the difference in the H3K4me3‐modified TRMT6 promoter region between TNBC and normal breast cells. F) Mechanism diagram of HMTs and OICR‐9429 regulating H3K4me3. G) The mRNA levels of TRMT6 in different concentrations of OICR‐9429 24 h treated MDA‐MB‐231 and Hs578T cells were measured by RT‐qPCR. H) The TRMT6 and H3K4me3 protein levels were measured via western blot assay after OICR‐9429 treatment (70 µm) for 48 h. I) ChIP‐qPCR was used to determine the enrichment of H3K4me3 at the promoter of TRMT6 in control or OICR‐9429 treatment (70 µm) MDA‐MB‐231 and Hs578T cells. J) The correlation of WDR5 and TRMT6 expression in 27 pairs of TNBC tissues. K) The mRNA change of WDR5 and TRMT6 after WDR5 was inhibited using siRNA in TNBC cells. L) The WDR5, TRMT6, and H3K4me3 protein levels were measured via western blotting assay after WDR5 suppression. M) ChIP‐qPCR was used to determine the enrichment of H3K4me3 at the promoter of TRMT6 in control or WDR5 deficiency TNBC cells. Significant differences were shown by ^*^ *p* <0.05; ^**^ *p* <0.01; ^***^ *p* <0.001.

To discover the potential regulatory mechanisms underlying the high TRMT6 expression in TNBC, we first retrieved ATAC‐seq and DNase‐seq datasets from the Encyclopedia of DNA Elements (ENCODE) database. The analysis revealed that the promoter region of TRMT6 (chr20:5950034‐5952533) in breast tissue samples exhibited prominent peaks and contained candidate cis‐regulatory elements (cCREs). These peaks and cCREs were characteristic of open chromatin regions, which suggested that the TRMT6 promoter region possesses active transcriptional regulatory potential (**Figure** **4**A). Subsequently, we analyzed visualized data from breast tissues in the National Institutes of Health Roadmap Epigenomics Project to examine the enrichment of critical histone modifications in the TRMT6 promoter region (Figure 4B). The results showed that among the transcription activation‐related modifications, H3K4me3 exhibited the highest peak, while other modifications with H3K4me1, H3K9ac, and H3K36me3 also displayed certain enrichment peaks. In contrast, modifications associated with transcriptional repression, H3K9me3 and H3K27me3, exhibited minimal to no detectable enrichment (Figure 4C). These findings preliminarily suggested that H3K4me3 may play a core regulatory role in the transcriptional activation of TRMT6. To verify the applicability of the above conclusions at the cellular level, we retrieved data from the Cistrome DB database, and found that the TRMT6 promoter region also exhibited highly enriched peaks of H3K4me3 in various breast cancer cell lines such as MDA‐MB‐231 and MB468 (Figure 4D). Then, we utilized the Chromatin Immunoprecipitation (ChIP) sequencing data with the highest H3K4me3 peak in the TRMT6 promoter region of the MDA‐MB‐231 cell line from the Cristom BD database, and employed HOMER to predict potential motifs and relevant transcription factors, including Lymphoid enhancer‐binding factor (LRF) and Transcription factor AP‐2 gamma (TFAP2C) (Figure S2A, Supporting Information). Next, we used ChIP‐qPCR assays to confirm that global H3K4me3 modification of TRMT6's promoter region was higher in TNBC cells than in normal mammary epithelial cells (Figure 4E). We then validated the regulatory effect of H3K4me3 on TRMT6 using OICR‐9429, a small‐molecule inhibitor that blocks H3K4me3 formation by preventing the WD repeat domain 5 (WDR5) interaction site on histone methyltransferases (HMTs) from binding to the H3 tail (Figure 4F). TNBC cells treated with different concentrations of OICR‐9429 for 24 h showed a dose‐dependent decrease in TRMT6 mRNA levels, prolonged treatment with OICR‐9429 for 48 h also remarkably downregulated H3K4me3 and TRMT6 protein levels (Figure 4G,H, Supporting Information). ChIP‐qPCR further confirmed OICR‐9429 treatment reduced H3K4me3 enrichment in the TRMT6 promoter region, directly confirming H3K4me3 regulates TRMT6 expression (Figure 4I).

Next, we focused on identifying the key HMT complex subunit that regulated the methylation of H3K4me3. The HMTs that catalyze H3K4me3 modification have SET domain‐containing protein 1A/B (SET1A/B) and lysine methyltransferase 2A/B (KMT2A/B, members of the MLL family) as their core catalytic subunits. Meanwhile, these HMTs also require WDR5, retinoblastoma binding protein 5 (RbBP5), Ash2L‐like histone lysine methyltransferase complex subunit (Ash2L), and DPY30 homolog (DPY30) as key auxiliary subunits, which together form the SET1/MLL complex.^[^ ^16^, ^17^ ^]^ First, we analyzed the correlation between TRMT6 and core components of the HMT complex using GEPIA2. While WDR5, RbBP5, Ash2L, and DPY30 showed strong positive correlations with TRMT6 in BC, while SET1A/B and KMT2A/B had weak correlations (Figure S2B, Supporting Information). Using MammOnc‐DB, we confirmed that these three strongly correlated genes (WDR5, RbBP5, DPY30) are highly expressed in TNBC (Figure S2C, Supporting Information). However, their associations with OS in TNBC patients differed, no OS shortening was observed in patients with high RbBP5, Ash2L, or DPY30 expression, whereas only high WDR5 expression correlated with poorer OS in TNBC patients (Figure S2D, Supporting Information). Meanwhile, siRNA‐mediated knockdown of RbBP5, Ash2L, or DPY30 in MDA‐MB‐231 and Hs578T cells did not fully reduce TRMT6 mRNA levels, suggesting these subunits were not key independent regulators of TRMT6 expression and may require synergy with other molecules to affect TRMT6. Importantly, WDR5 knockdown simultaneously reduced TRMT6 mRNA levels in both MDA‐MB‐231 and Hs578T cells (Figure S2E, Supporting Information; Figure 4K). Subsequently, we conducted western blotting experiments and found consistent experimental results (Figure S2F, Supporting Information).

Then, we comprehensively investigated whether WDR5 could serve as a cancer marker, and explored its relationship with TRMT6 in TNBC. We performed a correlation analysis and found a positive correlation between WDR5 and TRMT6 in TNBC samples collected from our cohort, which was similar to the correlation trend in the public database (Figure 4J). To verify whether WDR5 regulated TRMT6 protein expression, further western blotting experiments revealed that siRNA‐mediated WDR5 knockdown in MDA‐MB‐231 and Hs578T cells simultaneously suppressed TRMT6 protein level (Figure 4L). ChIP‐qPCR results also indicated that suppression of WDR5 remarkably reduced H3K4me3 enrichment at the TRMT6 promoter region, suggesting that WDR5 may regulate TRMT6 expression through histone modification (Figure 4J). We also detected the elevated mRNA expression of WDR5 in 27 pairs of clinical TNBC tissues compared with adjacent normal counterparts to clarify the clinical characteristics of WDR5 in TNBC (Figure S2G, Supporting Information). Consistently, western blotting confirmed higher WDR5 protein expression in TNBC tissues (Figure S2H, Supporting Information). These findings provide mechanistic evidence that TRMT6 overexpression in TNBC is regulated by WDR5‐mediated H3K4me3 modification.

### 2.5 TRMT6 Controls m^1^A tRNA Modification and Global Translation in TNBC

**Figure 5:**
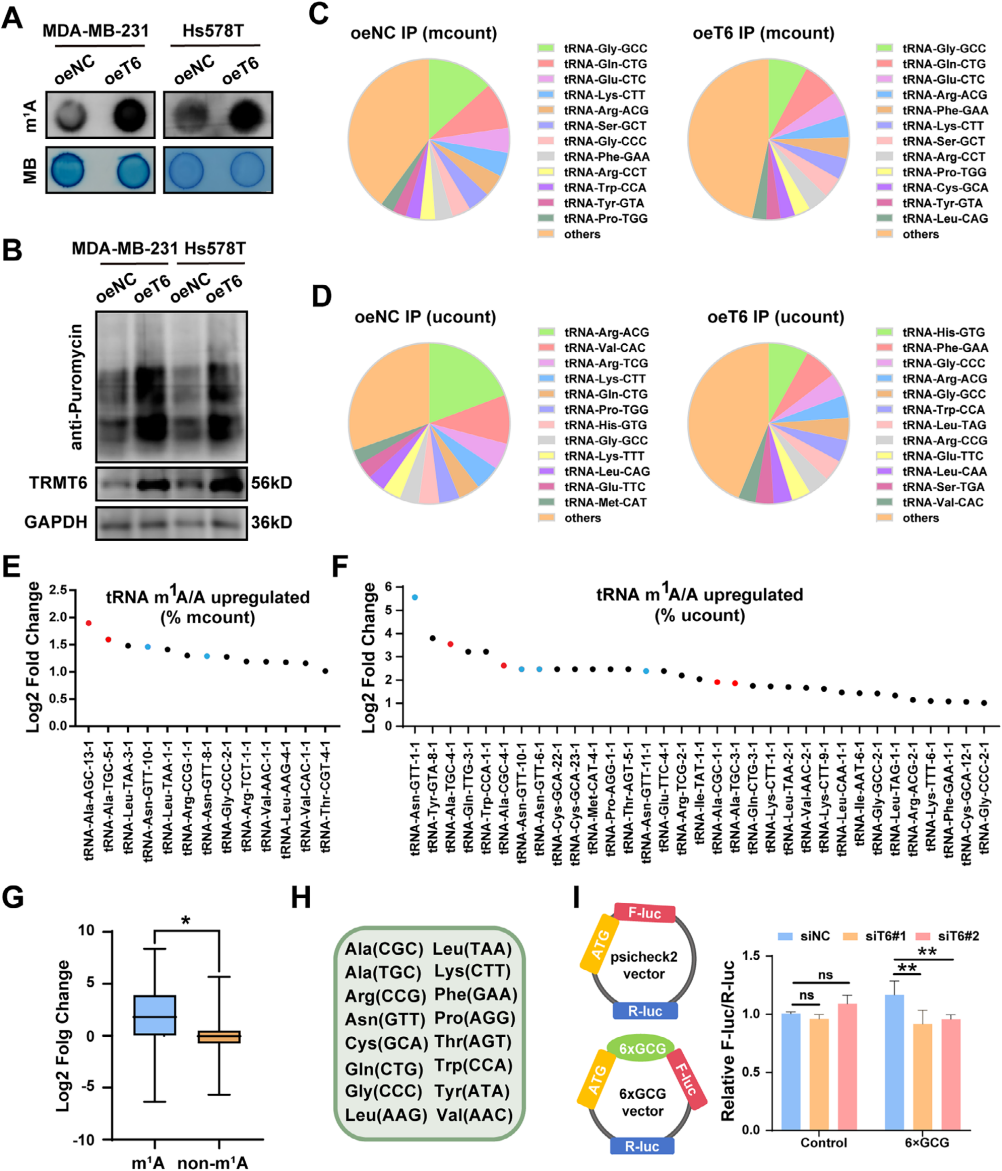
TRMT6 enhances m^1^A tRNA methylation levels and global mRNA translation. A) The dot blot experiment presents the effect of TRMT6 overexpression on the m^1^A modification level in RNA, with methylene blue staining as the sample control. B) Measurement of puromycin intake in MDA‐MB‐231 and Hs578T cells overexpressing wild‐type compared to control. C,D) The distribution pie chart shows the proportion of tRNA species obtained through IP (immunoprecipitation) in the oeNC (left) and oeT6 (right) groups of multi‐mapped (C) and uniquely mapped (D). E,F) The scatter plot shows the upregulation of tRNA m^1^A/A (% mcount) (E), (% ucount) (F), reflecting the differences in m^1^A modification levels among different tRNAs. G) Quantitative comparison of fold change in expression between m^1^A and non‐m^1^A tRNAs. H) List of m^1^A modified upregulated tRNAs identified by m^1^A‐tRNA‐MeRIP‐seq in MDA‐MB‐231 cells. I) Experimental results of dual luciferase reporter gene. Comparison of relative luciferase activity between Control group and 6‐GCG group under different interference conditions (siNC, siT6#1, siT6#2). Significant differences were shown by ^*^ *p* <0.05, ^**^ *p* <0.01, and ^***^ *p* <0.001, and ns, not significant.

Since TRMT6 has been proved as a m^1^A tRNA methyltransferase, we investigated the relationship between TRMT6‐mediated m^1^A modification and tRNA levels in TNBC. Dot blot assays confirmed increased m^1^A modification on RNA in the TRMT6 overexpression group (**Figure** **5**A). Meanwhile, puromycin assays demonstrated enhanced nascent protein synthesis in the TRMT6 overexpression group, indicating that TRMT6 could promote protein synthesis in TNBC cells (Figure 5B). These findings strongly support the crucial role of TRMT6 overexpression in promoting global m^1^A modification and enhanced translation. Emerging evidence indicates that TRMT6‐mediated m^1^A hypermethylation predominantly occurs in tRNAs.^[^ ^13^, ^18^ ^]^ Moreover, m^1^A modification on tRNAs maintains tRNA stability and promotes the translation process.^[^ ^19^, ^20^ ^]^ To explore the impact of m^1^A modification on specific tRNAs, we performed m^1^A‐tRNA‐MeRIP‐Seq in MDA‐MB‐231 cells. The sequence results were divided into two parts: uniquely mapped counts overlapping tRNA (ucount) and multi‐map‐corrected counts overlapping tRNA (mcount). We first demonstrated the difference between the composition of tRNAs enriched by the anti‐m^1^A antibody and those in the total RNA input (Figure 5C,D; Figure S3A,B, Supporting Information). In the IP (mcount) data, a high proportion of tRNAs, including GlyGCC, GlnCTG, and GluCTC, were clearly observed in both oeNC and oeT6 groups. Analysis of m^1^A tRNA modification between the oeNC group and oeT6 group indicated that high TRMT6 expression increased the abundance of m^1^A in specific tRNAs, such as AlaCGC and AsnGTT (Figure 5E,F). Statistical analysis revealed that TRMT6 overexpression induced significant increases in m^1^A‐modified tRNA levels, with non‐m^1^A‐modified tRNAs showing minimal expression changes (Figure 5G). In general, 16 tRNAs, including tRNA Ala and tRNA Asn exhibited higher m^1^A modification in the TRMT6 overexpression group (Figure 5H). Subsequently, MeRIP‐qPCR experiments were conducted to verify the upregulation of m^1^A modification in certain tRNAs (Figure S3C, Supporting Information). The elevated m^1^A signals in m^1^A‐modified tRNAs further validated the effect of TRMT6 overexpression (Figure S4D, Supporting Information). Moreover, high expression of TRMT6 reduced the m^1^A abundance in tRNA MetCAT (Figure S3E–G, Supporting Information). Additionally, dual‐luciferase assays confirmed that knocking down TRMT6 decreased the translation efficiency of the GCG codon by reducing m^1^A modification on tRNA AlaCGC (Figure 5I). Collectively, our data demonstrates that TRMT6 mediates m^1^A tRNA modification and influences the translation of specific codons.

### 2.6 TRMT6 Regulates the Translation of Anti‐Oncogenic mRNAs in m^1^A tRNA Decoded Codon‐Dependent Way

**Figure 6:**
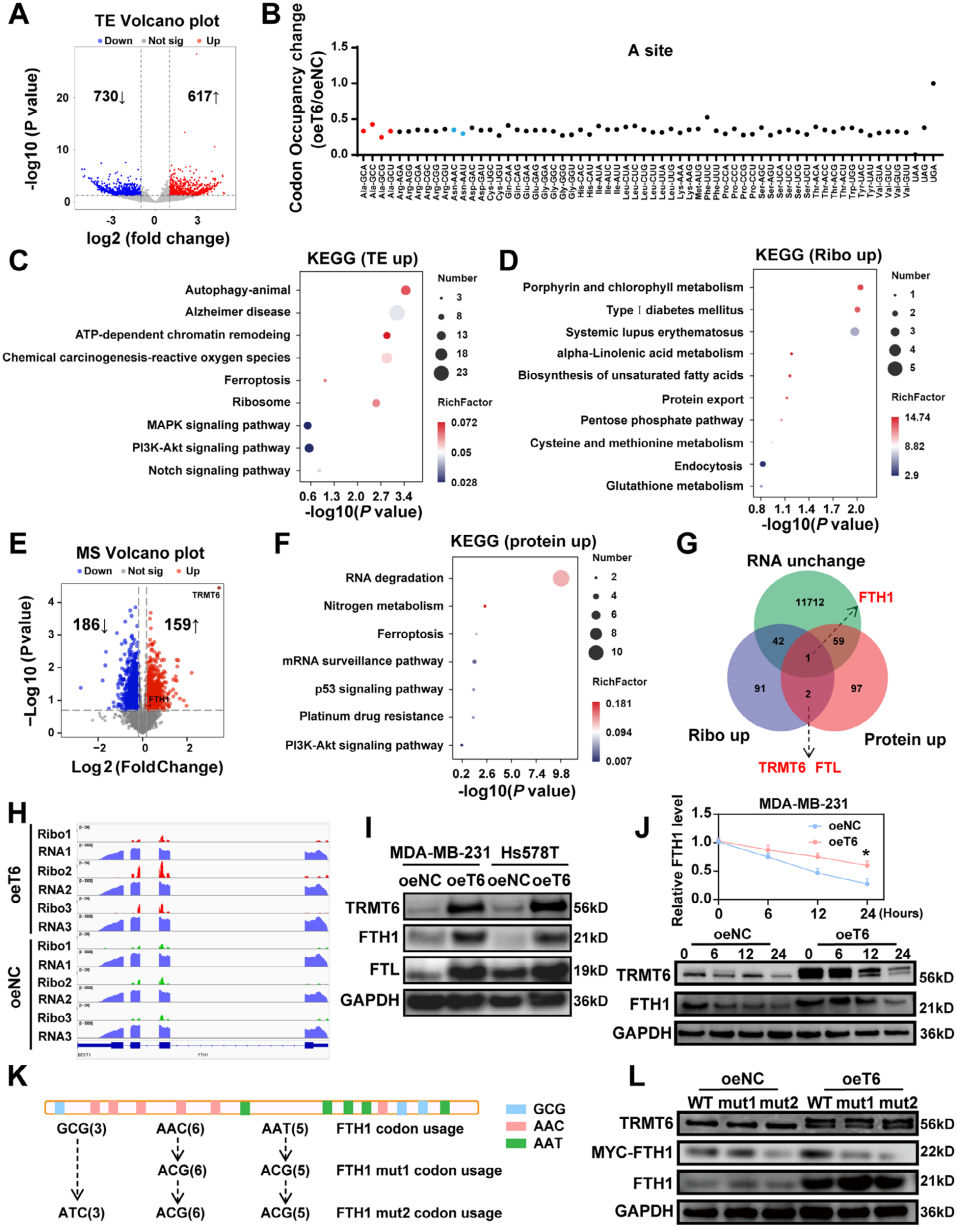
TRMT6 overexpression selectively modulates the translational efficiency of specific transcripts. A) Volcano Plot shows the TE up‐changed genes in TRMT6 overexpressed cells compared with wild‐type cells. B) Ribosome occupancy at individual codons at A sites. Red: codons decoded by m^1^A‐modified tRNAs‐Ala. Blue: codons decoded by m^1^A‐modified tRNAs‐Asn. The data are presented as mean. C,D) KEGG pathway analysis of TE up‐changed (C), Ribo up‐changed (D) genes in TRMT6 overexpressed cells compared with wild‐type cells. E) Volcano Plot shows the protein up‐changed genes in TRMT6 overexpressed cells compared with wild‐type cells. F) KEGG pathway analysis of protein up‐changed genes in TRMT6 overexpressed cells compared with wild‐type cells. G) Multi‐omics analysis identified FTH1 as a downstream target of TRMT6. H) IGV tracks for FTH1 from RNA‐seq and Ribo‐seq data in wild‐type and TRMT6 over‐expressed cells. Biological triplicates were analyzed. I) Western blotting showing FTL and FTH1 protein‐up expression relative to TRMT6 over‐expression. J) Detection of FTH1 degradation rate using CHX with 100 µg mL^−1^. K) Schematic diagram of the FTH1 codon‐switch assay. L) Western blotting analysis of FTH1‐MYC, FTH1, TRMT6, GAPDH protein expression in MDA‐MB‐231 wild‐type and TRMT6 over‐expression cells. GAPDH is used as a reference protein. Significant differences were shown by ^*^ *p* <0.05, ^**^ *p* <0.01, and ^***^ *p* <0.001, and ns, not significant.

To identify the downstream mRNA targets of TRMT6‐mediated m^1^A tRNA modification, we performed ribosome profiling sequencing (Ribo‐seq) and RNA sequencing (RNA‐seq) on TRMT6‐overexpressed cells and control MDA‐MB‐231 cells. Results showed robust changes in mRNA level and translation efficiencies (TEs), with many mRNAs exhibiting increased TEs upon TRMT6 overexpression (Figure S4A, Supporting Information). Our findings revealed significant alterations in TEs due to TRMT6 overexpression, with TEs increased in 617 mRNAs and decreased in 730 mRNAs (**Figure** **6**A). Although the biological significance of this observed codon pair bias remains unclear, it may impact translational efficiency by regulating the codon occupancy of ribosomal A and P sites. A site codon occupancy showed a general decrease in oeT6 cells from the Ribo‐seq data, with obvious reductions for AlaGCA, GCC, GCG, and GCU codons, as well as AsnAAC and AAU codons (Figure 6B). These codons are decoded by down‐regulated tRNAAlaCGC and tRNAAsnGTT. Concurrently, P site occupancy for AlaGCA, GCG, GCU codons, and AsnAAC, AAU codons was also decreased in oeT6 cells (Figure S4F, Supporting Information).

For targeted analysis of m^1^A‐modified tRNA translation efficiency, we focused on genes upregulated in TE for downstream analysis. Kyoto Encyclopedia of Genes and Genomes (KEGG) pathway analysis based on upregulated TEs and Ribo‐seq data revealed that enhanced mRNA translation was closely associated with iron metabolism, cell proliferation, and differentiation pathways, confirming the observed changes in overall cell growth and death (Figure 6C,D). Meanwhile, Gene Ontology (GO) enrichment analysis consistently linked upregulated TEs and Ribo‐seq genes to ferroptosis and cell proliferation processes (Figure S4B,C, Supporting Information). Moreover, we applied 4D Data‐Independent Acquisition (4D‐DIA) to detect the quantitative expression levels of proteins. Proteomics analysis showed only mild upregulation of protein levels for many TE‐upregulated genes (including ferroptosis related protein) (Figure 6E). KEGG and GO pathway analyses of upregulated proteins genes similarly confirmed regulation of ferroptosis and cell proliferation pathways (Figure 6F; Figure S4D, Supporting Information). We detected the protein expression of several ferroptosis‐related pathway molecules, including Transferrin Receptor (TRFC), Solute Carrier Family 7 Member 11 (SLC7A11), Glutathione Peroxidase 4 (GPX4), and Acyl‐CoA Synthetase Long Chain Family Member 4 (ACSL4), to verify that overexpression of TRMT6 inhibited TNBC ferroptosis (Figure S4E, Supporting Information). To focus on the effect of tRNA m^1^A modification in translation, we selected Ferritin Heavy Chain 1 (FTH1), a gene with increased translation and protein levels but unchanged RNA levels, for further analysis (Figure 6G). FTL and FTH1 are two subunits composing ferritin, and they maintain iron homeostasis and participate in various physiological and pathological processes through synergistic effects.^[^ ^21^, ^22^ ^]^ IGV visualization of FTH1 expression in Ribo‐seq and RNA‐seq confirmed this pattern, and RT‐qPCR showed no change in FTH1 RNA levels in oeT6 cells (Figure 6H; Figure S4G, Supporting Information). Western blotting validated protein expression of TRMT6, FTH1, and FTL in oeT6 cells (Figure 6I). Under TRMT6 overexpression, the cycloheximide (CHX) chase assay showed that the half‐life of FTH1 protein was prolonged, which clearly indicated a reduction in its degradation (Figure 6J). To determine whether TRMT6 regulates FTH1 mRNA translation via m^1^A modified tRNA, we generated FTH1 mutant cDNAs based on the frequency of FTH1 codon usage (Figure S4H, Supporting Information). One mutant (FTH1 mut1) replaced AAC, AAT codons with ACG, while another (FTH1 mut2) replaced GCG codons with ATC and AAC, AAT codons with ACG, based on FTH1 codon usage (Figure 6K). After stable transfection of equal amounts of WT or mutant FTH1 cDNAs (mut1 and mut2) into MDA‐MB‐231 cells with stably overexpressing TRMT6, western blotting showed altered FTH1 expression in mut1 and mut2 transfected cells (Figure 6L). Collectively, TRMT6 overexpression promotes TNBC progression by enhancing FTH1 translation through m^1^A tRNA decoded codon‐dependent manner.

### 2.7 TRMT6 Rescues RSL3‐Induced Cellular Ferroptosis in TNBC

**Figure 7:**
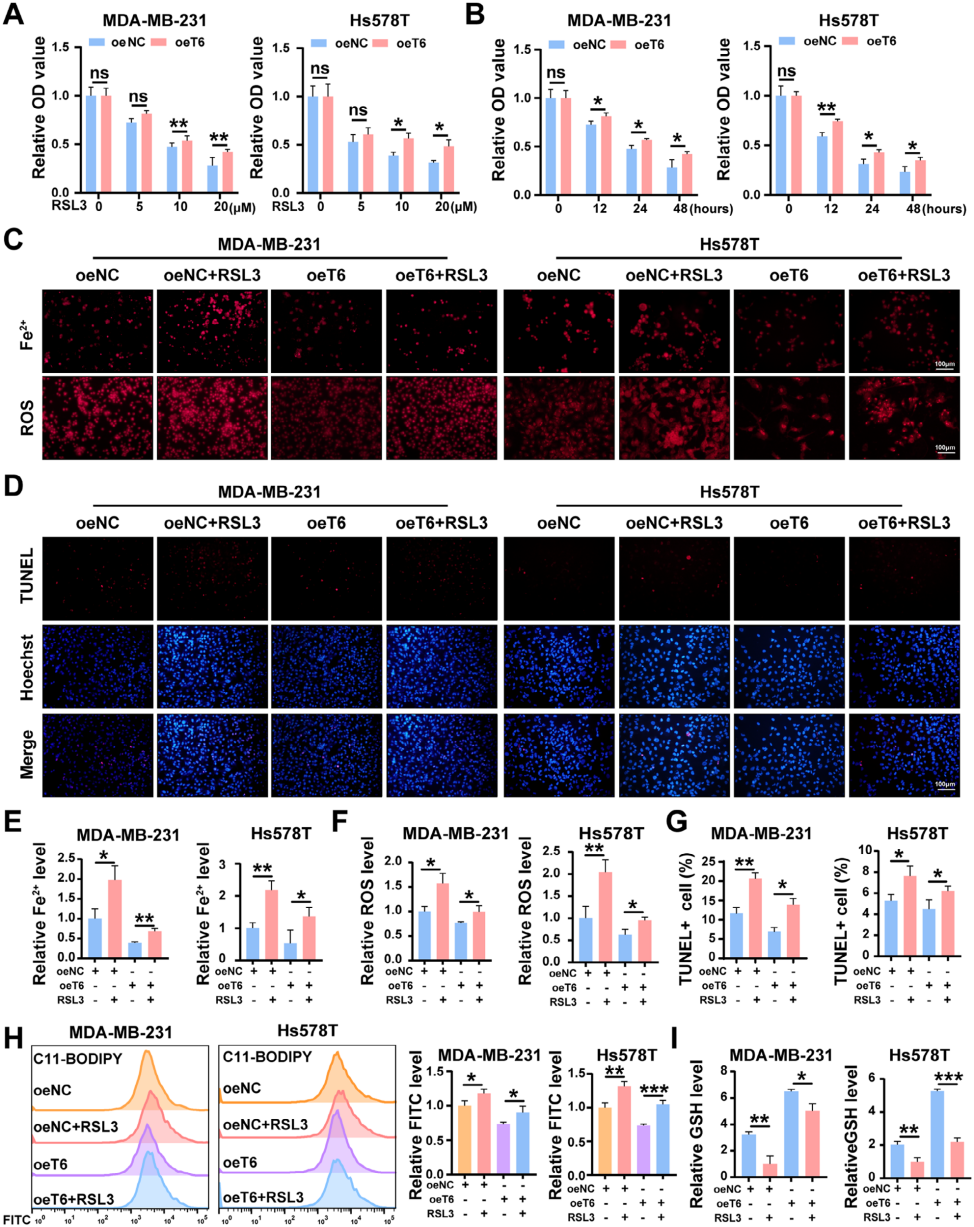
TRMT6 upregulation resists ferroptosis induced by RSL3. A) Indicated TNBC cells were incubated with different concentrations of RSL3 for 24 h and cell viability was assayed by CCK‐8 assay. B) Indicated TNBC cells were incubated with the same concentration of RSL3 for different times and cell viability was assayed by CCK‐8 assay. C) Intracellular Fe^2+^ detected by FerroOrange and the levels of ROS in TNBC cell lines under different experimental conditions. D) The apoptosis functions of the cells in different treatment groups were evaluated by TUNEL assay. E) Relative Fe^2+^ intensity per cell is shown. F) Relative ROS intensity per cell is shown. G) The statistical bar charts of TUNEL‐positive cells are presented. H) Flow cytometry was used to detect the fluorescence intensity of FITC after C11BODIPY staining. I) Microplate reader‐based detection of intracellular GSH. Scale bar = 100 µm. Significant differences were shown by ^*^ *p* <0.05, ^**^ *p* <0.01, and ^***^ *p* <0.001, and ns, not significant.

We next explored the correlation between TRMT6 and the ferroptosis pathway in TNBC malignant development. Previous studies have shown that RSL3, a ferroptosis agonist, increases intracellular Fe^2+^ and induces ferroptosis.^[^ ^23^ ^]^ To evaluate the therapeutic efficacy of RSL3 in TNBC cells, we treated the cells with different concentrations of RSL3 for 24 h and monitored cell viability at various time points. Subsequently, cell viability detected by the CCK‐8 assays showed that RSL3 induced cell death in a concentration and time‐dependent manner, while TRMT6 overexpression alleviated RSL3‐induced cell apoptosis (**Figure** **7**A,B). This indicated that TRMT6 conferred resistance to RSL3‐mediated ferroptosis in TNBC cells. Intracellular Fe^2+^ fluorescence staining and reactive oxygen species (ROS) staining experiments demonstrated that TRMT6 overexpression could rescue the RSL3‐induced abnormal elevation of Fe^2+^ and ROS (Figure 7C,E,F). The TUNEL experiment yielded similar results, as RSL3 reversed the apoptotic inhibitory effect of TRMT6 overexpression (Figure 7D,G). Consistent with these findings, flow cytometric analysis using C11BODIPY 581/591, a lipid peroxidation sensor, demonstrated that TRMT6 overexpression reduced RSL3‐induced lipid ROS accumulation in TNBC cells (Figure 7H). Detection of intracellular reduced glutathione (GSH) showed high levels in the TRMT6 overexpressing group, whereas RSL3 treatment decreased GSH levels (Figure 7I). We further demonstrated that knockdown of TRMT6 increased the cellular sensitivity to RSL3 and accelerated the process of ferroptosis (Figure S5A–I, Supporting Information). These experiments provided a theoretical basis for TRMT6 overexpression suppresses RSL3‐mediated ferroptotic cell death.

### 2.8 TRMT6 Accelerates Malignant Progression by Upregulating FTH1 Translation

**Figure 8:**
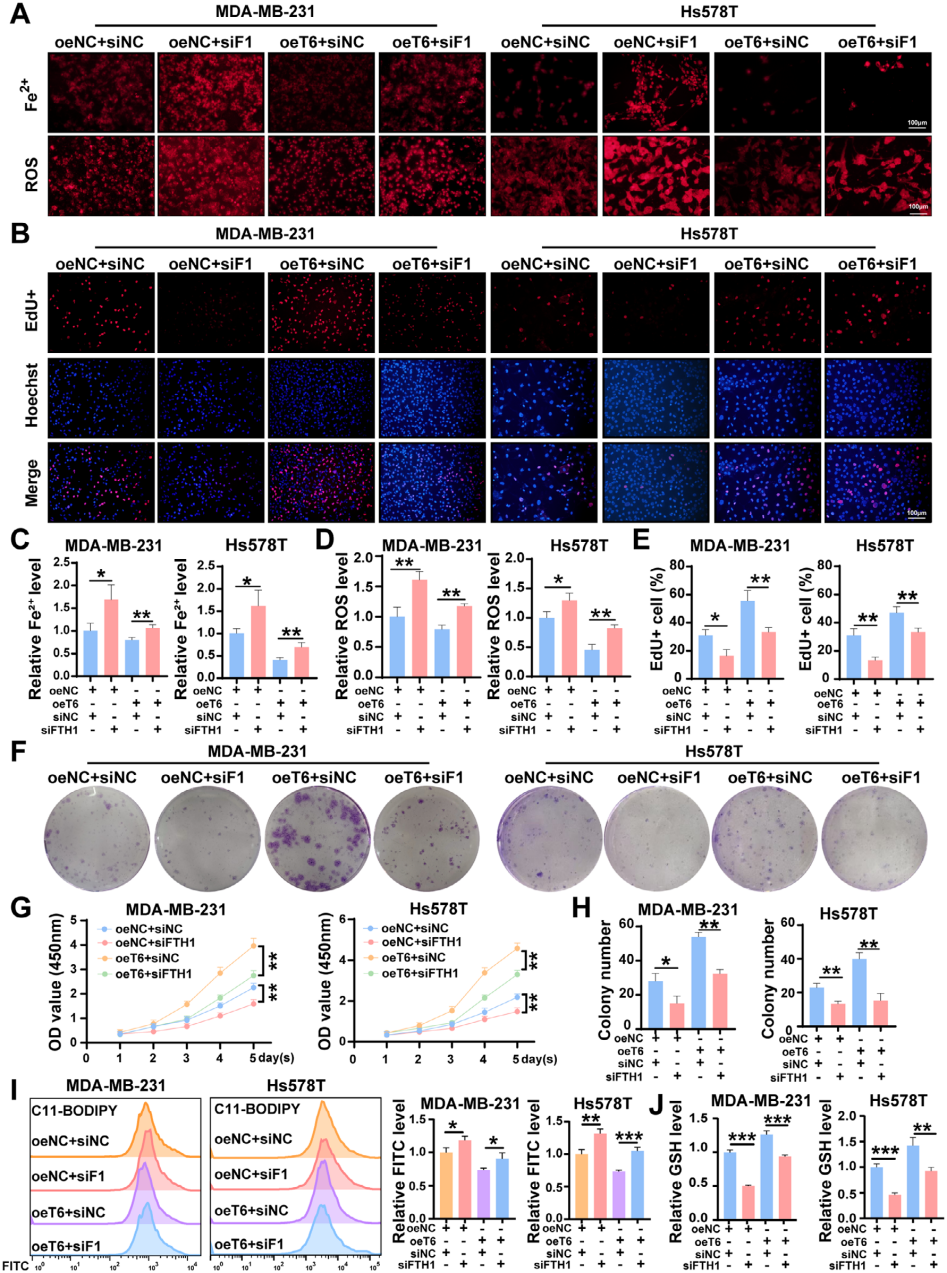
TRMT6 promotes TNBC malignant phenotypes through FTH1. A) Intracellular Fe^2+^ detected by FerroOrange and the levels of ROS in TNBC cell lines under different experimental conditions. B) EdU assays to evaluate changes in TNBC cell proliferation. C) Relative Fe^2+^intensity per cell is shown. D) The statistical bar charts of ROS level are displayed. E) The statistical bar charts of EdU^+^ cells are displayed. F) Evaluation of the effect of TRMT6 over‐expression and FTH1 knockdown on colony ability. G) CCK‐8 to assess the effects of TRMT6 over‐expression and FTH1 knockdown on the cell proliferation ability of MDA‐MB‐231 and Hs578T cells. H) The statistical bar charts of colony numbers are displayed. I) The fluorescence intensity of FITC was detected by flow cytometry after C11BODIPY staining. J) Detection of intracellular GSH. Scale bar = 100 µm. Significant differences were shown by ^*^ *p* <0.05, ^**^ *p* <0.01, and ^***^ *p* <0.001.

Given that TRMT6 overexpression enhances FTH1 translation, we sought to investigate whether FTH1 modulates TRMT6‐mediated inhibition of ferroptosis and tumor‐promoting functions. Through western blotting, siRNA knockdown of FTH1 in wild‐type and oeT6 cells was validated (Figure S6A, Supporting Information). When assessing Fe^2+^ and ROS levels, we found that FTH1 depletion increased intracellular Fe^2+^ levels and fluorescence intensity, partially rescuing tumor progression induced by TRMT6 overexpression and accelerating ferroptosis (**Figure** **8**A,C,D). Consistently, malondialdehyde (MDA) assays further confirmed the same trend of increased lipid peroxidation, with elevated MDA levels observed in FTH1‐depleted cells (Figure S6B, Supporting Information). EdU, colony formation, and CCK‐8 assays further indicated that silencing FTH1 markedly reduced the proliferation‐promoting effect of TRMT6, whereas TUNEL staining revealed increased apoptosis in these cells (Figure 8B,E–H; Figure S6C,E, Supporting Information). Similarly, C11BODIPY staining demonstrated heightened lipid peroxidation, while GSH detection revealed reduced intracellular levels of reduced glutathione in FTH1‐depleted cells, providing additional evidence for ferroptosis acceleration (Figure 8I,J). To evaluate the metastatic potential, Transwell assays demonstrated that FTH1 knockdown impaired migration, invasion, counteracting TRMT6's oncogenic effects (Figure S6D,F, Supporting Information). These results collectively demonstrate that FTH1 modulates the ferroptosis pathway and partially attenuates the tumor‐promoting effects induced by TRMT6 overexpression, thereby establishing FTH1 as a critical functional mediator of TRMT6 in the oncogenesis of TNBC.

To investigate the role of TRMT6 in normal breast epithelial cells, we overexpressed TRMT6 in MCF‐10A cells to observe corresponding cell phenotypes, while simultaneously knocking down FTH1 to examine phenotypes associated with the ferroptosis pathway. In the normal MCF‐10A cell line, we first performed EdU proliferation assay, TUNEL apoptosis detection, cell cycle analysis, and colony formation assays following TRMT6 overexpression (Figure S7A–H, Supporting Information). The results demonstrated that TRMT6 overexpression promoted the proliferative capacity of MCF‐10A cells, while markedly inhibiting cell apoptosis. The cell cycle distribution showed an increased proportion of cells in the S phase, and both the number and size of cell colonies were greater than those in the control group. These findings suggested that TRMT6 exerts proliferation‐promoting and anti‐apoptotic effects in MCF‐10A cells. On this basis, we further knocked down FTH1 in TRMT6‐overexpressing MCF‐10A cells and detected a series of ferroptosis‐related indicators (Figure S7I, Supporting Information). Compared with cells with only TRMT6 overexpression, cells with concurrent FTH1 knockdown exhibited marked increases of ROS and intracellular Fe^2+^ content (Figure S7J,K, Supporting Information); enhanced fluorescence intensity as revealed by C11BODIPY staining, a specific probe for lipid peroxidation, indicating aggravated lipid peroxidation (Figure S7L,M, Supporting Information); higher MDA content compared with the control group (Figure S7N, Supporting Information); and a notable reduction in GSH levels (Figure S7O, Supporting Information). These results indicated that FTH1 knockdown could induce intracellular oxidative stress imbalance and promote the occurrence of ferroptosis‐related molecular events in TRMT6‐overexpressing normal MCF‐10A cells, suggesting that FTH1 may be involved in regulating the effect of TRMT6 on ferroptosis in normal breast epithelial cells.

### 2.9 RSL3 Suppresses TRMT6‐Mediated Tumor Growth In Vivo via Reversing Ferroptosis Resistance

**Figure 9:**
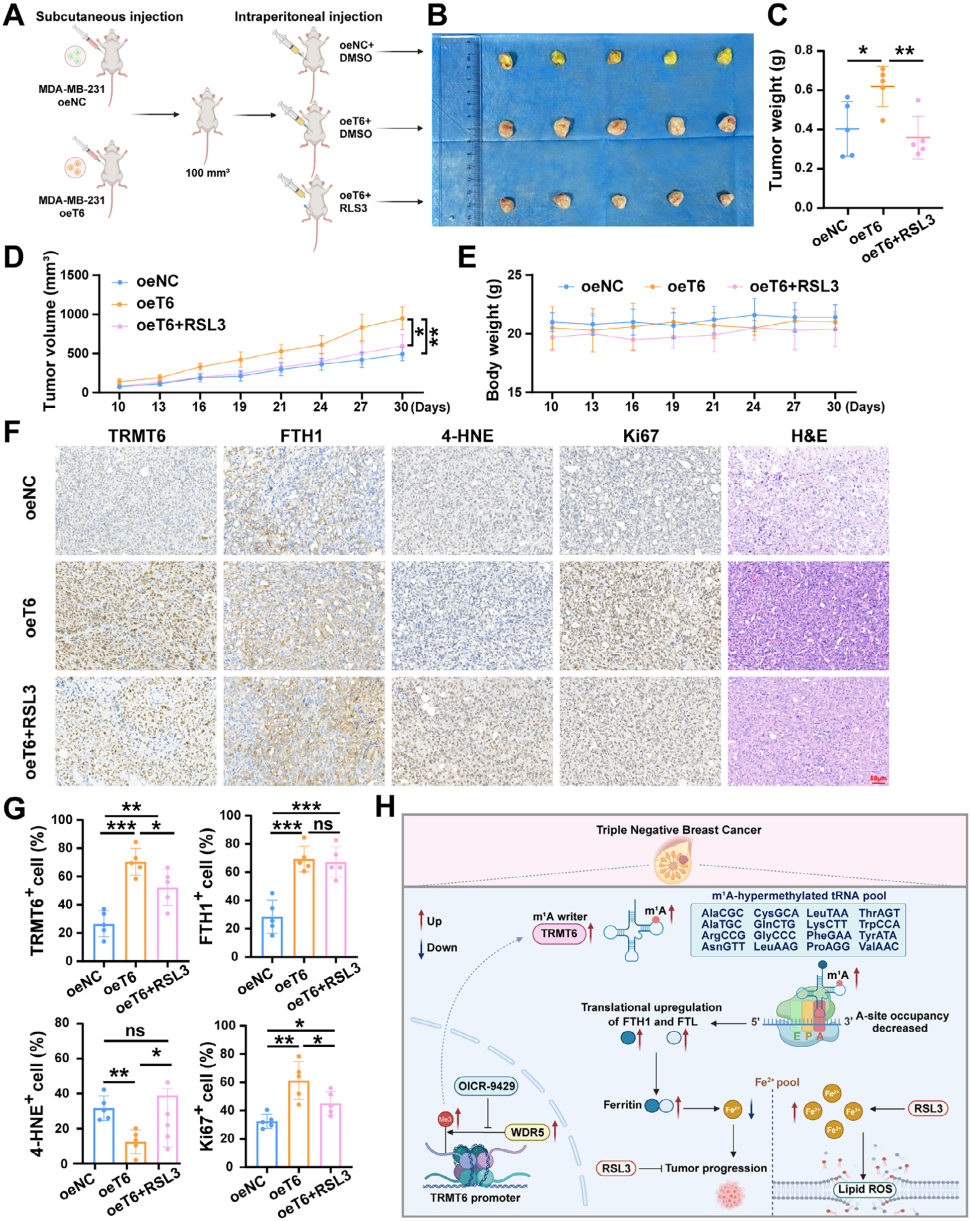
RSL3 eliminates TRMT6 induced tumor growth promotion in vivo. A) Schematic diagram to illustrate the treatment model protocol. B) Representative images of tumors in subcutaneous xenograft mice. C–E) Statistical analysis of tumor weight (C), body weight (D), and tumor volume (E) in different groups of mice (*n* = 5). Data were measured every three days. F) Representative IHC images of TRMT6, FTH1, 4‐HNE, Ki67 expression and H&E in serial segments of tumor tissue separated from the subcutaneous model. G) The quantitative statistical chart of IHC for TRMT6, FTH1, 4‐HNE, and Ki67. H) Schematic model illustrating the oncogenic role of TRMT6 in TNBC. The schematic was generated using Biorender. Scale bar = 50 µm. Significant differences were shown by ^*^ *p* <0.05, ^**^ *p* <0.01, and ^***^ *p* <0.001, ns, not significant.

To further clarify the in vivo role of TRMT6, we established animal xenograft models via subcutaneous injection of lentivirus‐transfected oeT6 cells and control cells. When ectopic subcutaneous tumor reached 100 mm^3^, the mice injected with oeT6 cells were randomly divided into two groups: one group received intraperitoneal injection of RSL3 (3 times per week for 3 weeks), while the remaining mice received an equal volume of vehicle (**Figure** **9**A). Compared with mice transfected with lentivirus TRMT6 overexpression, mice treated with empty lentivirus or injected with RSL3 exhibited slower tumor growth, and lentivirus TRMT6 overexpression was significantly greater in tumor weight and volume than the other two groups (Figure 9B–D, Supporting Information). None of the treatments‐RSL3, empty lentivirus alone, or lentivirus TRMT6 overexpression had noticeable effects on the mice's weight (Figure 9E). Hematoxylin and eosin (H&E) staining, TRMT6, FTH1, 4‐Hydroxynonenal (4‐HNE) and Ki67 immunohistochemistry of tissue sections from different treatment groups further supported these therapeutic effects (Figure 9F,G).

Concurrently, we established orthotopic xenograft models by inoculating lentivirus‐transfected oeT6 cells and control cells into the fourth mammary fat pads of immunodeficient NOD‐scid IL2Rγnull (NSG) mice, with the same three treatment groups: oeT6 group, oeT6 injected with RSL3 group, and empty vector control group (Figure S8A, Supporting Information). Monitoring of orthotopic tumors revealed consistent trends in tumor size, weight, and mouse body weight with the subcutaneous xenograft model. The oeT6 group exhibited the fastest tumor growth, with considerably greater tumor weight and volume than the other two groups, while RSL3 treatment effectively inhibited oeT6‐induced tumor proliferation (Figure S8B–D, Supporting Information). None of the treatments had noticeable effects on mouse body weight (Figure S8E, Supporting Information). Further assessment of tumor metastasis and invasion efficiency showed that the metastasis rate of orthotopic tumors in the oeT6 group was higher than that in the empty vector control group, and RSL3 treatment partially reversed this phenomenon. H&E staining of orthotopic tumor sections revealed a higher proportion of tumor capsule penetration in the oeT6 group, indicating enhanced invasiveness, whereas the RSL3‐treated group showed better capsule integrity compared to the oeT6 group (Figure S8F, Supporting Information). Immunohistochemical analysis further confirmed that the oeT6 group had increased expression of matrix metalloproteinase 9 (MMP9) and decreased expression of Epithelial Cadherin (E‐cadherin). RSL3 treatment downregulated MMP9 and upregulated E‐cadherin, consistent with the changes in tumor invasive and metastatic capabilities (Figure S8G, Supporting Information). To further assess the distant metastatic capacity, we harvested lung tissues from all experimental groups and performed H&E staining to identify and quantify lung metastatic nodules. Results revealed that the oeT6 group displayed a significantly higher number of lung metastatic nodules compared to the empty vector control group, confirming the enhanced metastatic potential induced by TRMT6. The reduction in lung metastatic nodules following RSL3 treatment corresponded to the suppression of invasive properties in our cell‐based assays, indicating a consistent anti‐metastatic phenotype (Figure S8H, Supporting Information). Together, these results indicate that the tumor‐promoting effect of TRMT6 in TNBC is at least partially regulated via the ferroptosis pathway.

In summary, our findings suggest that WDR5 transcriptionally activates TRMT6 by increasing H3K4me3 methylation at its promoter, and TRMT6 enhances FTH1 translation efficiency in an m^1^A tRNA‐decoding codon‐dependent manner, thereby inhibiting ferroptosis and promoting TNBC progression (Figure 9H).

## 3 Discussion

TNBC is one of the most aggressive subtypes of BC.^[^ ^24^, ^25^ ^]^ Here, we for the first time investigated the relationship between m^1^A tRNA methyltransferases and TNBC. We discovered that TRMT6 was highly expressed in TNBC and promoted tumorigenesis. Notably, WDR5 drove the transcription of TRMT6 via histone methylation. Subsequently, TRMT6 overexpression upregulated m^1^A methylation of tRNAs, thereby enhancing the translational efficiency and protein expression levels of downstream genes FTH1 and FTL. Importantly, the upregulated expression of FTH1 and FTL further enhanced iron ion storage within ferritin, suppressed ferroptosis and consequently drove the progression of TNBC. Therefore, our findings provide fresh insights into how m^1^A tRNA methyltransferases influence TNBC malignant development outcomes.

Translational regulation is crucial in tumor initiation and progression. Multiple RNA modifications participate in mRNA translation processes, encompassing both translation efficiency and accuracy, and they further modulate cancer development and progression.^[^ ^26^, ^27^ ^]^ Among them, m^1^A methylation mainly occurs on tRNA, especially m^1^A_58_ modification, which is closely related to the structural stability and correct folding of tRNA.^[^ ^28^, ^29^ ^]^ This modification is localized to specific nucleotide residues, as it has the capacity to disrupt Watson–Crick base pairing and impart a positive charge.^[^ ^30^ ^]^ Its positive charge further enables specific protein‐RNA and RNA‐RNA interactions, underscoring its multifaceted regulatory roles.^[^ ^31^ ^]^ Herein, we used m^1^A‐tRNA‐MeRIP sequencing to demonstrate the stable and high expression of m^1^A methylation modifications in three tRNAs: tRNA GlyGCC, GlnCTG, and GluCTC. Consistent with our findings, previous studies also reported robust m^1^A modification in these tRNAs.^[^ ^19^, ^20^ ^]^ Meanwhile, high expression of TRMT6 significantly increases the abundance of m^1^A in specific tRNAs, including AlaCGC and AsnGTT. Conversely, knockdown of TRMT6 was shown to markedly alter m^1^A modification levels on tRNA AlaTGC.^[^ ^32^ ^]^ Totally, the increase in m^1^A modifications of tRNAs ultimately leads to an upregulation of the translation efficiency of corresponding codons (e.g., GCG, which encodes Ala).

For seamless translation, tRNAs must be quickly accepted into the ribosomal A site and efficiently translocated to the P site.^[^ ^33^, ^34^ ^]^ Previous studies have shown that the occupancy of ribosomes at the A site is downregulated, indicating an increase in the translation efficiency of this codon.^[^ ^35^ ^]^ However, methylation modifications on tRNAs can affect the ribosomal A site occupancy. For instance, previous evidence indicated that the reduction of specific tRNA m^3^C methylation and ac^4^C modification increased the occupancy rate of the corresponding codon A site.^[^ ^36^, ^37^ ^]^ Our results showed that upregulation of m^1^A modification on tRNA leads to a general downregulation of A site ribosome occupancy, which is consistent with the upregulation of downstream gene translation efficiency. Therefore, multiple studies suggested that m^1^A modifications may affect wobble pairing with codons such as GCG by optimizing ribosome binding A site efficiency.

WDR5 is predominantly recognized for its role in histone‐lysine N‐methyltransferase 2 (KMT2) complexes, which facilitate transcription through H3K4 methylation.^[^ ^17^, ^38^ ^]^ Through the key interface of the interaction site (WIN motif), WDR5 selectively binds to members of the KMT2 enzyme family (such as mixed lineage leukemia 1, MLL1) to catalyze H3K4me3 methylation modification.^[^ ^39^, ^40^ ^]^ The small‐molecule antagonist OICR‐9429 disrupts WDR5‐MLL1 complex formation by binding to the WDR5 WIN site, attenuating H3K4me3 deposition at oncogenic enhancer elements.^[^ ^41^ ^]^ This compound has demonstrated potential as an anticancer agent in leukemia and some solid tumors, thus we investigated its ability in regulating histone methylation modifications and anti‐TNBC efficacy.^[^ ^41^, ^42^ ^]^ Our study demonstrated the high expression of WDR5 in TNBC and verified its regulatory correlation with TRMT6. Previous studies have also confirmed that WDR5 is highly expressed in TNBC and can serve as an independent prognostic indicator.^[^ ^43^ ^]^ Limitations exist in our study regarding the predicted motifs and transcription factors (ZEB1, PLAGL2) that are potentially related to H3K4me3 within the TRMT6 promoter region. Our prediction results are based on one sequencing data from a single cell line. Differences in chromatin accessibility across various cell types may lead to variations in the exposure of motifs. Even among sequencing data from the same cell line, there can be discrepancies in the length of peak fragments, which in turn affects the results of motif enrichment analysis and the predicted transcription factors. The value of this prediction lies in providing a clear direction for subsequent research, which requires further experimental exploration and validation.

Ferritin, a ubiquitously expressed iron storage protein in organisms, is composed of 24 subunits assembled into a hollow spherical structure. It comprises two types of subunits: the FTH1 and the FTL.^[^ ^44^ ^]^ Among these, FTH1 possesses iron oxidase activity, enabling it to catalyze the oxidation of ferrous ions (Fe^2+^) to ferric ions (Fe^3+^) and sequester them within the protein core. This function is pivotal for regulating intracellular iron homeostasis and mitigating oxidative stress. FTL primarily contributes to iron storage and mobilization, facilitating the formation and stabilization of iron cores within ferritin complexes.^[^ ^45^ ^]^ Currently, serum ferritin testing holds significant clinical relevance for diagnosing iron deficiency anemia, iron overload disorders, and assessing nutritional status. And iron can affect the heat production of fat cells. Mice iron metabolism disorder specifically in adipocytes have impaired thermogenesis, increased insulin resistance, and low‐grade inflammation.^[^ ^46^ ^]^ Meanwhile, silibinin disrupted the nuclear receptor coactivator 4‐FTH1 interaction and inhibited iron autophagy.^[^ ^47^ ^]^ Beyond its role in iron metabolism, ferritin serves as a tumor marker with clinical utility for diagnosing specific malignant tumors.^[^ ^48^ ^]^ Emerging evidence indicates that FTH1 is implicated in the pathophysiology of non‐metastatic breast cancer and Diffuse large B cell lymphoma.^[^ ^49^, ^50^ ^]^ In our study about TNBC, upregulation of TRMT6 can promote m^1^A tRNA modification, improve the translation efficiency of FTH1, and elevated both mRNA and protein expression of FTL. The latest research indicated that the newly discovered derivative of cryptotanshinone is positioned as a promising targeted therapeutic drug for FTH1, providing a strategy to combat cancer by inducing iron deficiency anemia in N2 type tumor‐associated neutrophils (TANs) and TNBC cells.^[^ ^25^ ^]^

Phenotypically, inhibition of the m^1^A methyltransferase TRMT6 and FTH1 noticeably reduced cell proliferation, highlighting their critical roles in tumor growth. Elevated WDR5 mRNA expression in most tumor types correlates with poor prognosis, underscoring its importance as a transcriptional regulatory hub. These three targets, TRMT6 (epigenetic modification), WDR5 (transcriptional regulation), and FTH1 (metabolic homeostasis), collectively form a multi‐dimensional regulatory network driving tumor progression. To optimize therapeutic strategies, future research should improve treatment protocols in multiple aspects. First, targeting m^1^A chemical marks or TRMT6 methyltransferase directly to regulate m^1^A modification levels on tRNA may emerge as a promising strategy for anticancer therapy. Currently, research teams have proposed using cholesterol‐conjugated siRNA to specifically target TRMT6, thereby inhibiting CRC tumor growth and metastasis.^[^ ^15^ ^]^ Second, exploring the combined application strategy of TRMT6 and WDR5 inhibitors can enhance antitumor efficacy through a dual inhibition mechanism. Third, establishing predictive models that integrate m^1^A modification levels, TRMT6 and WDR5 expression will enable precise patient stratification. Finally, the development of tissue‐specific delivery systems, such as nanocarriers responsive to the tumor microenvironment, can help mitigate systemic toxicity arising from the basal expression of WDR5 and TRMT6 in normal tissues. Collectively, these approaches provide a theoretical foundation for developing novel anticancer therapies based on tRNA modifications, offering potential breakthroughs for refractory tumors such as TNBC.

## 4 Conclusion

In summary, this study aimed to gain insight into the mechanisms by which aberrant m^1^A tRNA modifications promote the progression of TNBC. One significant finding of this study is that increased WDR5 expression in TNBC leads to elevated H3K4me3 methylation levels in the TRMT6 promoter region, thereby activating TRMT6 transcription. Furthermore, TRMT6 enhances the translation of FTH1 and FTL by regulating m^1^A modification of tRNAs, rendering TNBC cells resistant to ferroptosis. In vitro and in vivo experiments confirmed that treatment with RSL3 inhibits tumor growth by enhancing lethal lipid peroxidation and ferroptosis in TRMT6‐expressing cells. These results provide new insights into the regulatory mechanisms of ferroptosis and propose a promising strategy for TRMT6‐based therapy in patients with TNBC.

## 5 Experimental Section

### Clinical Specimens

TNBC specimens along with corresponding adjacent normal tissues were sourced from female breast cancer patients at the First Affiliated Hospital of Zhengzhou University. These patients had not undergone preoperative chemotherapy or radiotherapy. The adjacent tissues were collected from a standardized distance of 3 cm away from the BC tissues. The clinical characteristics of 27 paired TNBC patients are detailed in Table S1 (Supporting Information). Each participant provided written informed consent voluntarily.

### Cell Culture and Treatment

Human breast cancer cell lines (MDA‐MB‐415, MCF‐7, T47D, ZR‐75‐1, BT‐474, SK‐BR‐3, MDA‐MB‐231, Hs578T, BT‐549, MB436, HCC1143, CAL‐51, MB436, MB468, SUM149, and SUM159) were purchased from ATCC (USA), and the human normal mammary epithelial cell line (MCF10A) was obtained from Pricella (China). These cell lines were maintained in DMEM or RPMI 1640 medium (Gibco, China), enriched with 10% fetal bovine serum (FBS, BaiDi Biotechnology Co., Ltd) and 2% penicillin‐streptomycin solution (Biosharp, China). The cell culture plates were then incubated at 37 °C in a humidified environment containing 5% CO_2_. OICR‐9429 was purchased from Shandong Sparkjade Biotechnology Co., Ltd.

### Quantitative Reverse Transcriptase Polymerase Chain Reaction (RT‐qPCR)

Total RNA from cell lines was isolated using the SteadyPure RNA Extraction Kit (Accurate Biology, China), following the manufacturer's protocol. For breast tissue, the FastPure Complex Cell/Tissue Total RNA Isolation Kit (Vazyme, China) was utilized for RNA extraction. cDNA was synthesized with the Evo M‐MLV RT Premix for qPCR (Accurate Biology, China). RT‐qPCR assays were performed on an Applied Biosystems 7500 Real‐Time PCR System (Applied Biosystems, Foster City, CA, USA), using the Evo M‐MLV One Step RT‐qPCR Kit (SYBR) Ver.2 (Accurate Biology, China). The relative mRNA expression levels were calculated using the 2^‐ΔΔCt^ method, with GAPDH serving as the endogenous reference gene. The sequences of the primers used in RT‐qPCR are provided in Table S2 (Supporting Information).

### RNA Interference and Lentivirus‐Mediated Infection

Short interfering RNAs (siRNAs) directed at TRMT6 (designated siTRMT6#1 and siTRMT6#2) along with their corresponding controls (siNC) were obtained from RiboBio (Guangzhou, China). The transfection procedure employed the RiboFECT CP Transfection Kit. siRNAs targeting FTH1 and WDR5 (referred to as siFTH1 and siWDR5) together with control siRNAs (siNC) were procured from Sangon Biotech (Shanghai, China), with transfection carried out using the RNA Transmate reagent (Sangon Biotech, China). The coding sequence of TRMT6 (NC_000020.11), intended for stable transfection, was amplified and subsequently cloned into the pHBLV‐CMV‐MCS‐3FLAG‐EF1‐ZsGreen‐T2A‐PURO vector. Hanbio Biotechnology Co., Ltd. (Shanghai, China) constructed the lentiviral vectors incorporating TRMT6 overexpression and wild‐type TRMT6. Specific lentiviral vectors were designed, assembled, and packaged. MDA‐MB‐231 and Hs578T cells underwent stable transfection utilizing Polybrene (Biosharp, China) in combination with lentiviral solution (Hanbio, Shanghai). Cells that stably expressed the transfected genes were selected using a puromycin concentration of 10 µg mL^−1^ (Biosharp, China). The efficiency of transfection was assessed via RT‐qPCR or western blotting. The sequences of the siRNAs are presented in Table S3 (Supporting Information).

### Western Blotting

To lyse cells, RIPA buffer (Beyotime, China) with added protease inhibitors (Beyotime, China) was utilized. The BCA kit (NCM Biotech, China) was used to measure and normalize protein concentration. Protein lysates underwent separation via sodium dodecyl sulfate‐polyacrylamide gel electrophoresis (SDS‐PAGE) and were then transferred onto PVDF membranes (Millipore, USA). Bands were detected using ECL chemiluminescent reagents (Epizyme, China). The list of antibodies employed in this study is provided in Table S4 (Supporting Information).

### tRNA Dot Blot

First, total RNA was extracted using the Trizol method. The RNA was then subjected to gel electrophoresis, and the tRNA fraction was recovered by gel extraction. Then, the concentration of the extracted tRNA was determined to be 1500 ng µL^−1^. Next, the PVDF membrane was soaked in 0.1% Triton for 30 min and then dried by a drying oven. 1.5 µL of each tRNA sample was spotted onto the membrane, and the tRNA was immediately cross‐linked and fixed using 254 nm ultraviolet light for 10 min. Subsequently, the membrane was stained with methylene blue, photographed, washed, and then blocked. After overnight incubation with the anti‐m^1^A antibody (Abcam, UK), the membrane was washed, incubated with the Goat anti‐mouse antibody (Proteintech, China), and exposed for detection.

### Immunohistochemical (IHC) Staining and H&E Staining

TNBC tissue and mouse tumor tissue sections were deparaffinized, dehydrated, antigen‐retrieved, blocked, and incubated with primary antibodies. Staining was performed using diaminobenzidine (DAB) detection. H&E staining involved fixation, embedding, sectioning, deparaffinization, staining, dehydration, clearing, and mounting of tissue sections. IHC analysis was conducted using Image J software and adopted a blinded random selection based on morphological features. Protein levels of TRMT6, FTH1, 4‐HNE, and Ki67 were measured by calculating the average density of positive staining in five randomly selected fields of view.

### Functional Assays In Vitro

For cell viability, 3000 transfected MDA‐MB‐231 and Hs578T cells per well were seeded in 96‐well plates. Viability was measured at 0, 24, 48, 72, and 96 h using the Cell Counting Kit‐8 Reagent (APExBIO, Shanghai, China) per the manufacturer's protocol. In colony formation assays, 1000 cells per well were seeded in six‐well plates with fresh medium and incubated at 37 °C. After two weeks, colonies were stained with 0.5% crystal violet for counting. EdU assays used the Click‐iT EdU‐594 In Vitro Kit (Servicebio, Wuhan, China). Fluorescent signals were observed via inverted fluorescence microscopy, and EdU‐positive cell rates were analyzed with Image J. Transwell assays assessed cell migration and invasion. In these assays, 2×10⁵ transfected cells in 200 µL serum‐free medium were placed in the upper chamber (LABSELECT, China) with or without Matrigel (Corning, NY, USA). After 48 h, cells on the membrane's outer surface were fixed with 4% paraformaldehyde, stained, and counted with crystal violet to evaluate migration and invasion. Images were captured under an inverted fluorescence microscope, and the number of cells that migrated through the chamber was quantified using Image J. Cells were stained with FerroOrange (Servicebio, Wuhan, China), BODIPY 581/591 C11(ThermoFisher, China) and MitoSOX Red (ThermoFisher, China) to assess intracellular iron levels and oxidative stress, respectively. Briefly, cell culture medium was replaced with serum‐free medium containing 1 µM FerroOrange, 5 µM BODIPY 581/591 C11 and 10 µm MitoSOX Red, followed by incubation at 37 °C for 30 min in the dark. After three washes with PBS to remove excess probe, fluorescence intensity was immediately captured using a flow cytometer or inverted fluorescence microscope to reflect the levels of Fe^2+^, oxidized BODIPY‐C11, and ROS, respectively. For GSH determination, GSH standard solutions of different concentrations were prepared using a GSH assay kit (Servicebio, China). For sample preparation, cells were collected and added to pre‐cooled extraction buffer, sonicated, centrifuged at 8000 g at 4 °C for 10 min, and the supernatant was retained for use. For sample measurement, a 96‐well plate was taken, with 10 µL of standard solution or diluted extract added to each well. Then 150 µL of chromogenic working solution was added to each well, followed by incubation at 37 °C in the dark for 10 min. The absorbance was measured at 412 nm using a microplate reader, and a standard curve was plotted. The GSH content in the samples was calculated according to the standard curve. Collect cells into a centrifuge tube, add an appropriate amount of lysis buffer (Servicebio, China), sonicate, then centrifuge at 12 000 × g at 4 °C for 10 min, and transfer the supernatant to a new centrifuge tube. Dilute the MDA standard solution in gradients to prepare standard solutions of different concentrations. Take plastic tubes, add 50 µL of the sample to be tested or standard solution to each tube, then add 50 µL of thiobarbituric acid reagent and mix gently. Place the EP tubes in a boiling water bath for 30 min to allow MDA to fully react with TBA and form a red complex, then quickly cool to room temperature. Transfer 100 µL of the complex to a 96‐well plate and measure the absorbance at 532 nm using a microplate reader.

### Cell Cycle Analysis

To analyze the cell cycle, harvested cells were washed with cold PBS, then fixed using 70% ethanol at 4 °C for 12 h. Next, they were incubated with a Cell Cycle Detection Kit (KeyGEN, Nanjing, China), along with RNase and the DNA‐binding dye propidium iodide (PI) at 37 °C for 30 min. The cell cycle distribution was determined via flow cytometry using a BD Accuri C6 flow cytometer (BD Accuri C6, San Diego, CA, USA). The data obtained were further analyzed and visualized with FlowJo v.10 software (BD Biosciences, Franklin Lakes, NJ, USA).

### Cell Apoptosis Analysis

Upon collection, cells were washed in PBS and stained with the PE Annexin V Apoptosis Detection Kit I (BD Pharmingen), with the addition of Binding Buffer, Annexin V‐PE, and 7‐AAD, followed by incubation in the dark. Apoptosis detection was carried out using a flow cytometer (BD Accuri C6, San Diego, CA, USA), with data analysis performed using FlowJo v.10 software (BD Biosciences, Franklin Lakes, NJ, USA). For TUNEL staining to assess cell apoptosis, the TUNEL Cell Apoptosis Detection Kit (Servicebio, Wuhan, China) was used. Transfected cells were fixed with paraformaldehyde, incubated with TUNEL solution, and subsequently stained with DAPI. Images were captured with a fluorescence microscope, and the apoptosis rate was determined by analyzing the number of TUNEL‐positive cells using Image J software.

### ChIP Assays

The ChIP assays was conducted using the Beyotime (China) ChIP kit as per the manufacturer's guidelines. In summary, proteins and DNA were cross‐linked via 1% formaldehyde. Post‐cell lysis, chromatin was broken down into fragments employing micrococcal nuclease. Subsequently, immunoprecipitation of the DNA‐protein complexes took place in the presence of 5 µg of antibody. Reversal of the DNA‐protein cross‐links was accomplished through NaCl and proteinase K treatment. The DNA was then purified and recovered. Finally, the DNA underwent further analysis via RT‐qPCR. Fold enrichment was determined following normalization to 1% input.

### m^1^A‐tRNA‐MeRIP‐Seq

m^1^A‐tRNA‐MeRIP‐Seq technical services were provided by Aksomics (Shanghai, China). Briefly, total RNA was purified and quantified from cells. The Hydro‐tRNAseq method was used to isolate tRNAs from total RNA. After m^1^A and m^3^C demethylation, the tRNAs were partially hydrolyzed. Small RNA sequencing libraries constructed were from 19–35 nt tRNA fragments using the NEBNext Multiplex Small RNA Library Prep Set for Illumina. The libraries were quantified on an Agilent 2100 Bioanalyzer, denatured, diluted, and then loaded onto an Illumina sequencer for sequencing.

### Puromycin Incorporation Assays

To assay protein synthesis rates, cells were treated with 30 µm puromycin at 37 °C for 1 h, after which they were lysed for protein extraction. Western blotting with anti‐puromycin antibody (Kerafast, USA) was performed to assess puromycin incorporation.

### Ribo‐Seq

Cells were harvested after blocking ribosome translocation by flash freezing; ribosome profiling sequencing was performed by APExBIO (Shanghai, China). Cell lysates were digested with ribonuclease (RNase), and ribosome‐protected RNA fragments were collected by sucrose gradient ultracentrifugation or a column kit. rRNA was removed, and ribosome‐protected fragments (RPFs, ≈30 bp) were collected, which could be mapped back to the original mRNA to determine the exact positions of translating ribosomes. Sequencing was performed on an Illumina platform, followed by bioinformatics analysis. Differentially translated genes (DTGs) between groups were identified using FDR <0.05 and |log2FC| >1.

### Data‐Independent Acquisition Mass Spectrometry (DIA‐MS)

The experimental process mainly covered protein isolation, peptide break down, chromatographic separation, LC‐MS/MS analysis, DIA data collection, database search and analysis of qualitative and quantitative results, and bioinformatics analysis.

### Dual‐Luciferase Reporter Assay

In the luciferase reporter assay, plasmids from Sangon Biotech enabled the insertion of six repeated codons right after the firefly luciferase gene's ATG start codon in the psicheck2 vector. Transfection of these plasmids into cells was achieved using Lipofectamine 3000 (APExBIO, China). The Dual‐Glo Luciferase Assay System (Servicebio) measured firefly and renilla luciferase activities 48 h post‐transfection. Relative firefly luciferase activity, normalized to renilla luciferase activity, was compared across different groups.

### RNA m^1^A Dot Blot

For the m^1^A dot blot analysis, RNA was extracted and quantified, then diluted to a concentration of 1000 ng µL^−1^. After denaturation by heating at 95 °C for 5 min, 2 µL of the RNA solution was spotted onto PVDF membranes that had been treated with 0.2% Trixon (Amersham, GE Healthcare, USA). The membranes were cross‐linked using UV light, blocked with 5% skim milk, and incubated overnight at 4 °C with an m^1^A‐specific antibody. Subsequently, they were incubated with an enzyme‐conjugated secondary antibody. Finally, the membranes were visualized using a chemiluminescence system. Membranes stained with methylene blue (MB) served as controls.

### Animal Models

All animal experiments were performed in accordance with the Institutional Animal Care and Use Committee of the First Affiliated Hospital of Zhengzhou University. Female NSG mice (5 weeks old) were acquired from Beijing HFK Laboratory Animal Technology Co., Ltd. For in vivo xenograft models, mice were randomly grouped into three groups (*n* = 5). For the in vivo orthotopic tumor model, cells were injected into the mammary fat pad following a small skin incision, and the wound was sealed with tissue adhesive. Stably transfected MDA‐MB‐231 cells (oe‐NC, oe‐T6) were prepared as single‐cell suspensions and each mouse was subcutaneously injected with 5 × 10^6^ cells. Mice were euthanized thirty days post‐tumor inoculation. Ten days after inoculation, when tumor volume reached ≈100 mm^3^, mice were randomly divided into three groups (*n* = 5): control (LV‐NC + DMSO), oe‐TRMT6 (LV‐M1 + DMSO), and oe‐TRMT6 + RSL3 (LV‐M1 + RSL3). RSL3 was given by intraperitoneal injection at 10 mg kg^−1^, once a week for three doses, each 0.2 mL. It was first dissolved in DMSO, then diluted in water with 0.4% carboxymethylcellulose (Merck Millipore) and 0.2% Tween‐80 (Sigma). Tumor volume (length × width^2^ × 0.5, in mm^3^) was measured every three days during treatment, and tumor tissues were collected after three weeks.

### Statistical Analysis

All experimental results are expressed as mean ± Standard Deviation (S.D.). Statistical evaluations were conducted using SPSS 20.0 and GraphPad Prism software. The paired or unpaired Student's *t*‐test was applied to assess differences in expression between two groups. Spearman correlation analysis was conducted to explore the correlation between TRMT6 and WDR5. The *p*‐value of less than 0.05 was regarded as statistically significant.

### Ethical Approval and Participation Consent

All participants provided written informed consent, following the guidelines of the Declaration of Helsinki. Ethical approval was granted by the Ethics Committee of the First Affiliated Hospital of Zhengzhou University (2022‐KY‐0735). Animal experimental procedures were approved by the Experimental Animal Ethics Committee of Henan University of Chinese Medicine (DWLL202201015). All animal housing and experiments were conducted in strict accordance with the institutional guidelines for care and use of laboratory animals.

## Conflict of Interest

The authors declare no conflict of interest.

## Author Contributions

Y.L., X.W., and J.L. contributed equally to this work. Y.L. responsible for conceptualization, data curation, formal analysis, investigation, and writing. X.W. conducting investigation, methodology, validation, and writing. J.L. providing resources, investigation, data curation, and validation. Z.N. overseeing resources, project administration, and validation. J.H. performing investigation and data curation. Q.G. carrying out investigation, data analysis, validation, and writing. D.D. contributing investigation, methodology, and data visualization. Y.Z. conducting data curation and formal analysis. F.H. leading conceptualization, funding acquisition, and supervision. M.Z. managing resources, funding acquisition, and investigation. H.L. directing methodology, formal analysis, data interpretation, and supervision. J.H. overseeing conceptualization, resources, data curation, funding acquisition, project administration, and writing. J.L. guiding conceptualization, resources, supervision, funding acquisition, and writing.

## Supporting information



Supporting Information

## Data Availability

The data that support the findings of this study are available from the corresponding author upon reasonable request.
