# Supplementary material for: WDR5‐H3K4me3 Epigenetic Axis Promotes TRMT6‐Dependent tRNA M1A Modification to Facilitate Triple‐Negative Breast Cancer Progression by Suppressing Ferroptosis
Source: Adv Sci (Weinh). 2025 Dec 14;13(11):e13277. doi: 10.1002/advs.202513277 (PMC12931253; doi:10.1002/advs.202513277)
Supplement: Supplementary file 1 — Supporting Information [file ADVS-13-e13277-s001.pdf]

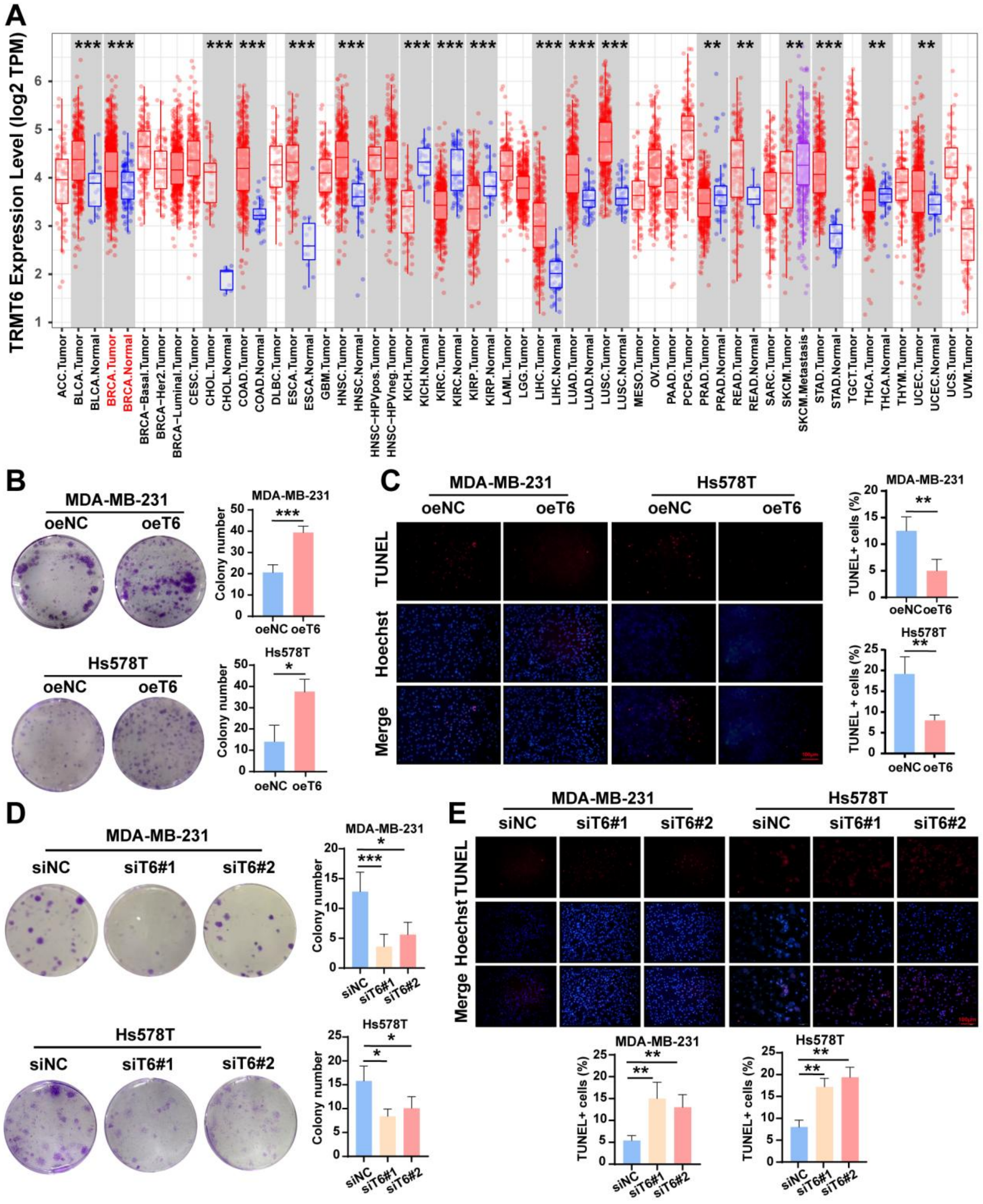

Supplementary Figure 1: TRMT6 is required for TNBC progression *in vitro*.

(A) TRMT6 expression levels in different cancer types analyzed by the TIMER database (http://timer.comp-genomics.org/). **(B)** Evaluation of the effect of TRMT6 over-expression on colony ability. **(C)** TUNEL assay used to assess apoptotic ability of cells after TRMT6 over-expression. **(D)** Evaluate the colony formation ability after TRMT6 knockdown. **(E)** TUNEL assay to examine the apoptosis in each group of cells.

*$P < 0.05$; **$P < 0.01$; ***$P < 0.001$. The *P* values were determined by Student's t test (unpaired two-tailed). All data are presented as mean ± S.D.

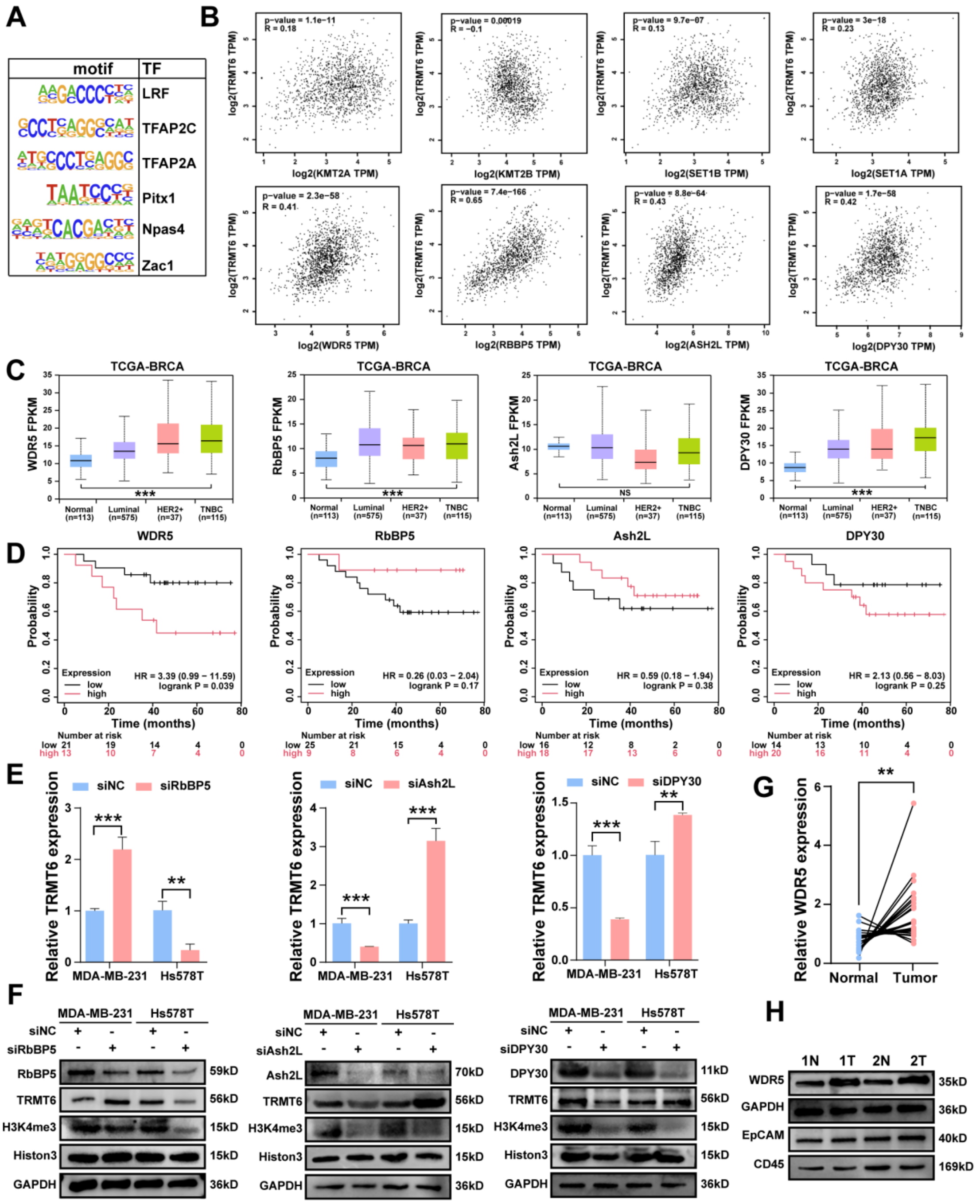

Supplementary Figure 2: WDR5 is the key regulator of TRMT6 among HMTs in TNBC.

(A) Prediction of motifs and TFs related to H3K4me3 modification in MDA-MB-231 cell line. **(B)** GEPIA2 analysis of the Spearman correlation analysis of TRMT6 and HMTs mRNA expression level in BRCA tissues samples from TCGA-GTEx database. **(C)** The expression of HMTs in TNBC patience from TCGA database in MammOnc

website (https://resource.path.uab.edu/MammOnc-Home.html). **(D)** Correlation between HMTs expression and overall survival of TNBC patients according to the Kaplan-Meier Plotter (https://kmplot.com/analysis/). **(E)** The mRNA level of TRMT6 after siRbBP5, DPY30, Ash2L. **(F)** The protein level of TRMT6, and H3K4me3 after siRbBP5, DPY30, Ash2L. **(G)** The mRNA expression of WDR5 in 27 pairs of TNBC tissues. **(H)** The protein level of WDR5 in two pairs of human TNBC specimens. N: Normal, T: Tumor. *$P < 0.05$, **$P < 0.01$, ***$P < 0.001$. The data are presented as mean ± S.D.

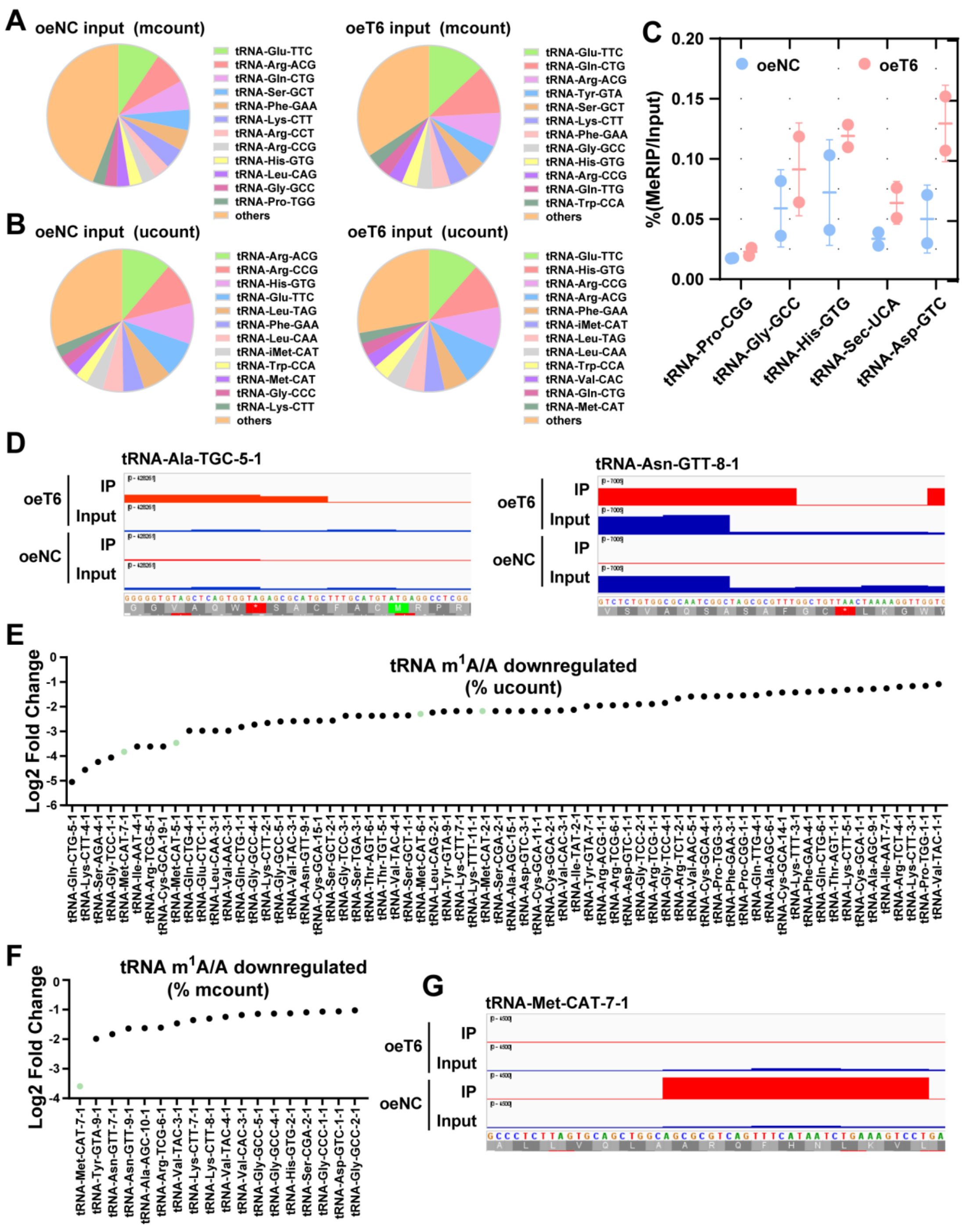

Supplementary Figure 3: TRMT6 regulates m^{1}A modification level in different kinds of tRNA.

(A-B) Pie chart showing the identified top 12 individual tRNA in oeNC and oeT6 groups of input tRNA mcount **(A)** and input tRNA ucount **(B)**. **(C)** m^{1}A methylation percentage in input tRNA between oeNC and oeT6 groups by MeRIP-qPCR. The data

are presented as mean ± S.D. **(D)** Representative images from Integrative genome viewer (IGV) displaying different IP/Input at the motif sequence of indicated tRNA. **(E-F)** The scatter plot shows the downregulation of tRNA m^1A/A (% ucount) **(E)**, (% mcount) **(F)**. The data are presented as mean. **(G)** IGV displaying different IP/Input at the motif sequence of tRNA-Met.

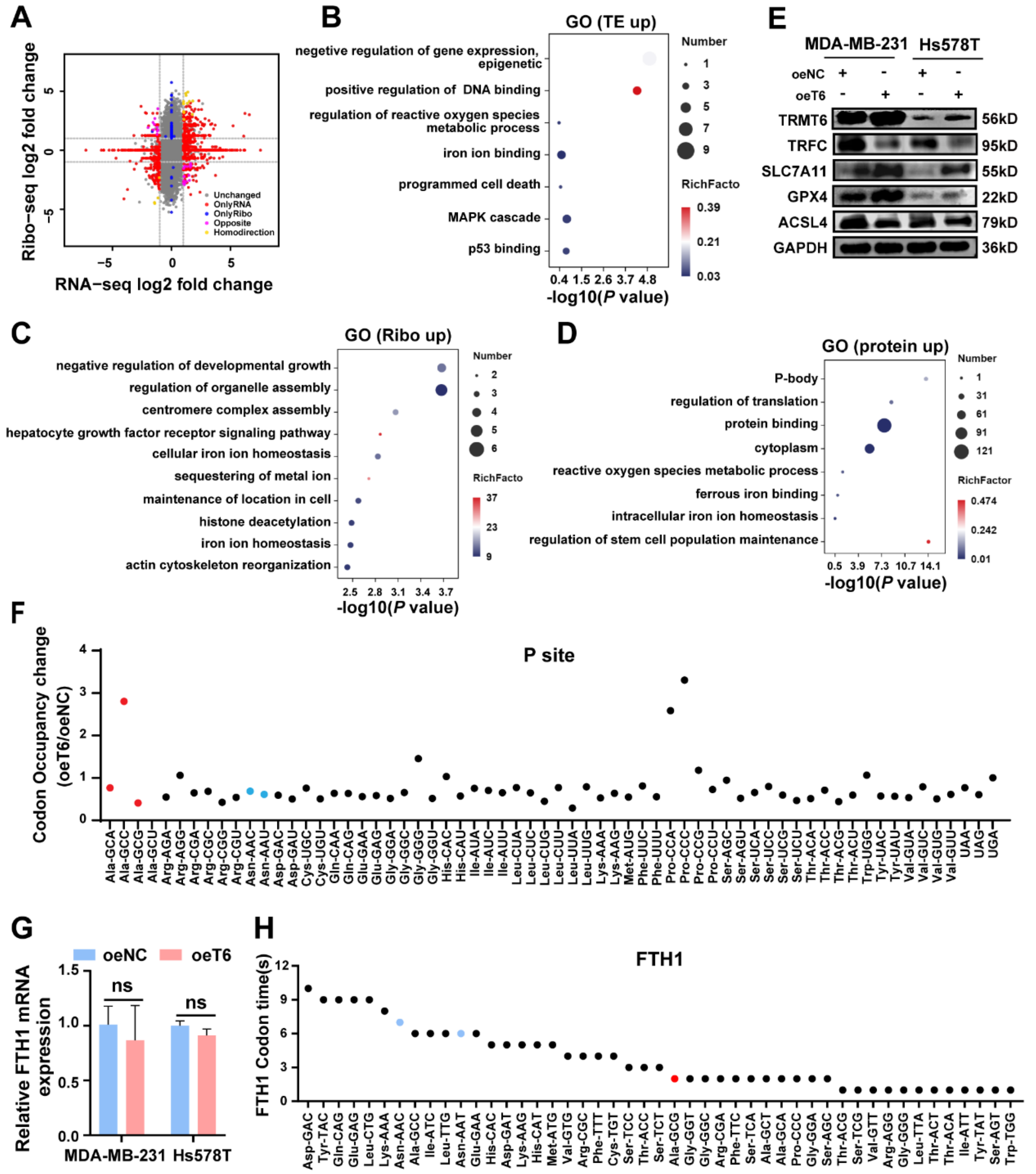

Supplementary Figure 4: Ribo-seq and DIA-MS revealed upregulation of translation efficiency in TRMT6 overexpressing cells.

(A) The four-quadrant plot reflecting the relationship between Ribo-seq and mRNA-seq. **(B-D)** Gene Ontology analysis of TE-up **(B)**, Ribo-up **(C)** and protein-up genes **(D)** upon TRMT6 overexpression. (**E**) Protein level changes of genes involved in the ferroptosis pathway upon TRMT6 overexpression. **(F)** Ribosome occupancy at

individual codons at P sites. Red: codons decoded by m^1A-modified tRNAs-Ala. Blue: codons decoded by m^1A-modified tRNAs-Asn. The data are presented as mean. **(G)** RT-qPCR analysis of relative FTH1 levels after over-expression of TRMT6 and wild-type in TNBC cells. The data are presented as mean ± S.D. ns, not significant. **(H)** The number of FTH1 codons times are used. Red: codons decoded by m^1A-modified tRNAs-Ala. Blue: codons decoded by m^1A-modified tRNAs-Asn. The data are presented as mean.

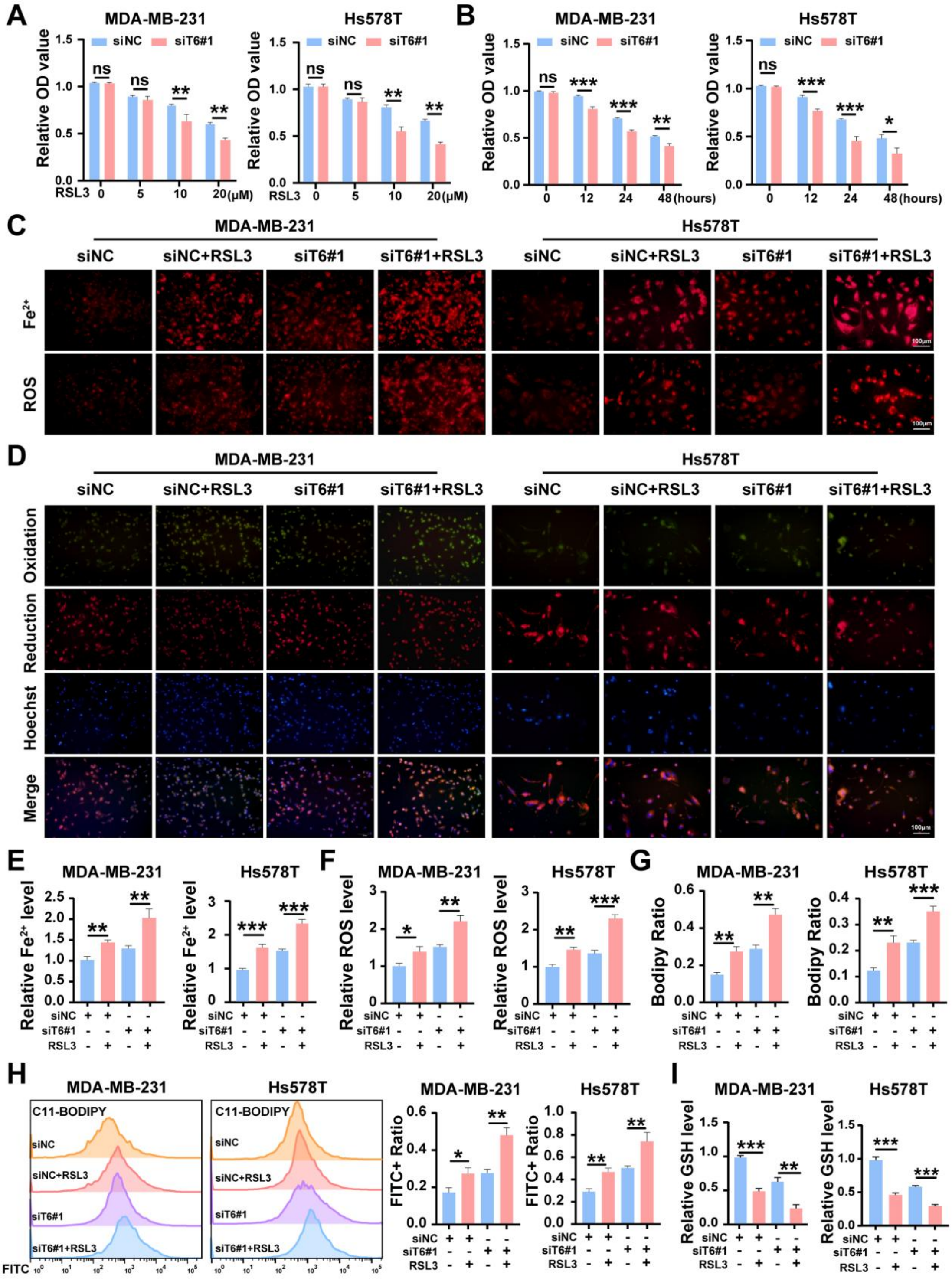

Supplementary Figure 5: TRMT6-mediated phenotypic changes correlate with the ferroptosis pathway.

(A) The CCK-8 assay showed the viability of TNBC cells after 24h incubation with different concentrations of RSL3. **(B)** CCK-8 measures TNBC cell viability after

TRMT6 knockdown with RSL3 treatment over different time. **(C)** Intracellular Fe^{2+} detected by FerroOrange and the levels of ROS in TNBC cell lines under different experimental conditions. **(D)** Fluorescent imaging after C11 BODIPY staining. **(E)** Relative Fe^{2+} intensity per cell is shown. **(F)** Relative ROS intensity per cell is shown. **(G)** The statistical bar charts of oxidation are presented. **(H)** Flow cytometry was used to detect the fluorescence intensity of FITC after C11BODIPY staining. **(I)** Microplate reader-based detection of intracellular GSH. Scale bar = 100 μm. Significant differences were shown by $*P < 0.05$, $**P < 0.01$, and $***P < 0.001$, and ns, not significant.

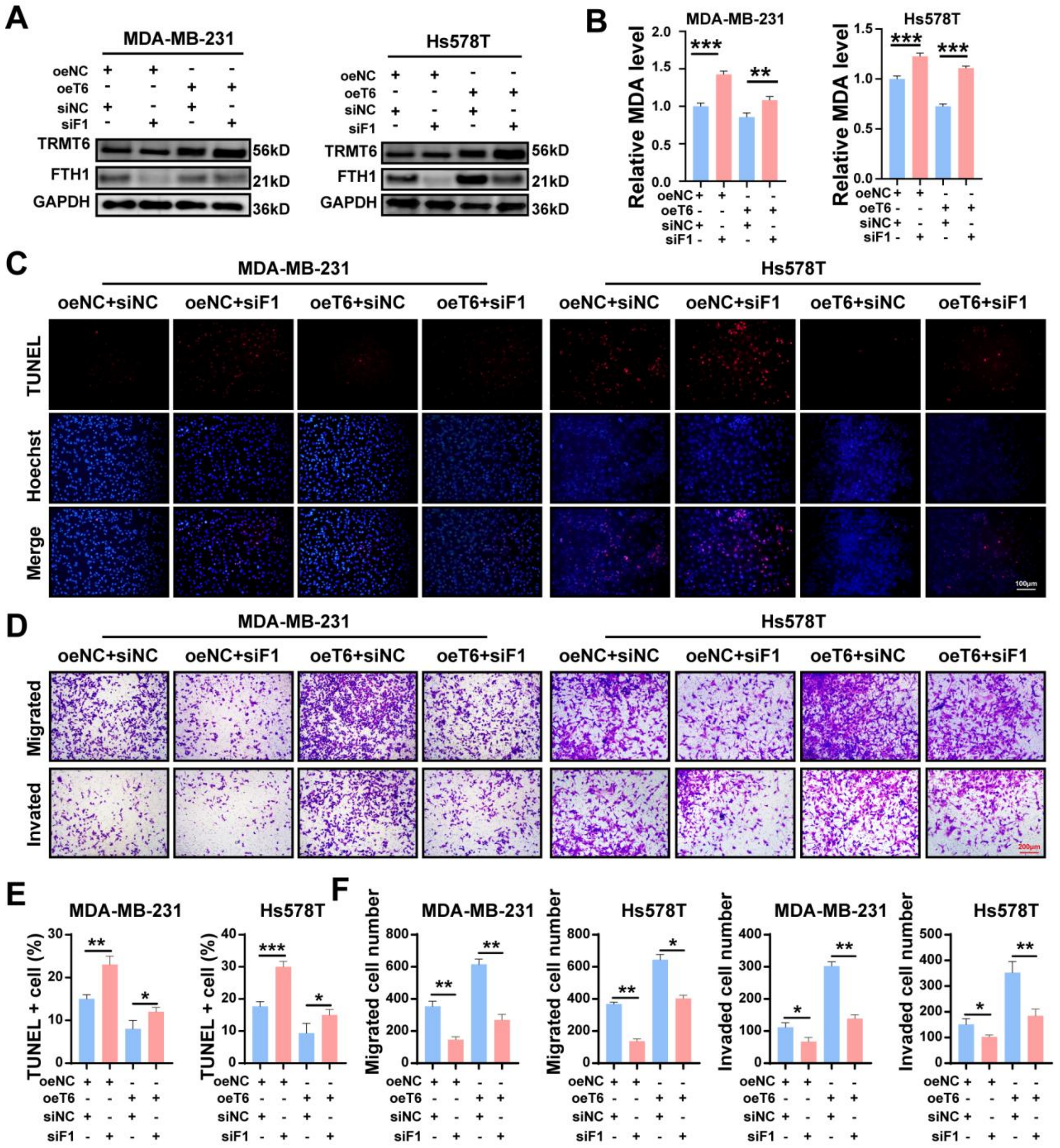

Supplementary Figure 6: Knocking down FTH1 reverses TRMT6 mediated iron death resistance in TNBC.

(A) Western blotting validation of FTH1 knockdown after overexpression of TRMT6 in MDA-MB-231 and Hs578T cells. **(B)** MDA assay reflects the degree of intracellular lipid oxidative damage. **(C)** TUNEL assay assesses cell apoptotic ability. **(D)** Transwell assays to profile cell migration and invasion capacity. **(E)** The statistical bar charts of TUNEL positive cells are displayed. **(F)** The statistical bar charts of migrated cell

number are displayed. The data are presented as mean ± S.D. Scale bar = 100 μm. *$P < 0.05$, **$P < 0.01$, ***$P < 0.001$, and ns, nonsignificant.

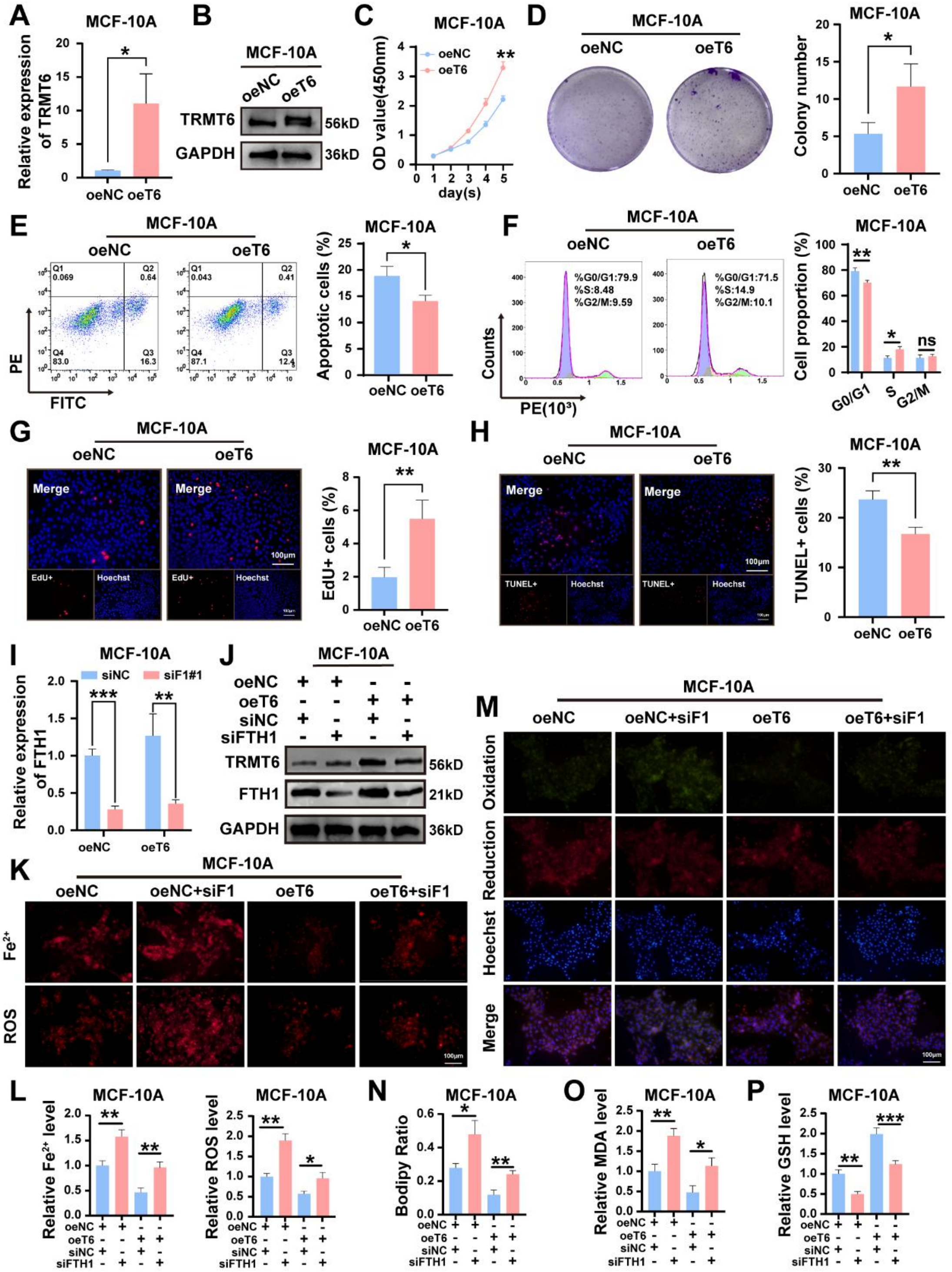

Supplementary Figure 7: Overexpression of TRMT6 promotes MCF-10A *in vitro*.

(A) RT-qPCR analysis of relative TRMT6 levels after over-expression of TRMT6 and wild-type in MCF-10A cells. **(B)** Western blotting confirmation of TRMT6 in indicated MCF-10A cells. **(C)** CCK-8 was used to assess the effects of TRMT6 overexpression

on the cell proliferation ability of MCF-10A cells. **(D)** Evaluation of the effect of TRMT6 over-expression on colony ability. **(E)** Flow cytometric analysis of apoptotic cells percentage. **(F)** Flow cytometric analysis of cell cycle distribution. **(G)** EdU assays to evaluate changes in MCF-10A proliferation. **(H)** TUNEL assays to evaluate changes in MCF-10A apoptosis. **(I)** The bar chart shows FTH1 mRNA levels. **(J)** Western blotting confirmation of siFTH1 in MCF-10A cells. **(K)** Intracellular Fe^{2+} detected by FerroOrange and the levels of ROS in MCF-10A under different experimental conditions. **(L)** Relative Fe^{2+} and ROS intensity per cell is shown. **(M)** Fluorescent imaging after C11 BODIPY staining in MCF-10A. **(N)** The statistical bar charts of oxidation are presented. **(O)** MDA assay reflects the degree of intracellular lipid oxidative damage. **(P)** Microplate reader-based detection of intracellular GSH. Scale bar = 100 μm. Significant differences were shown by $^{*}P < 0.05$, $^{**}P < 0.01$, and $^{***}P < 0.001$.

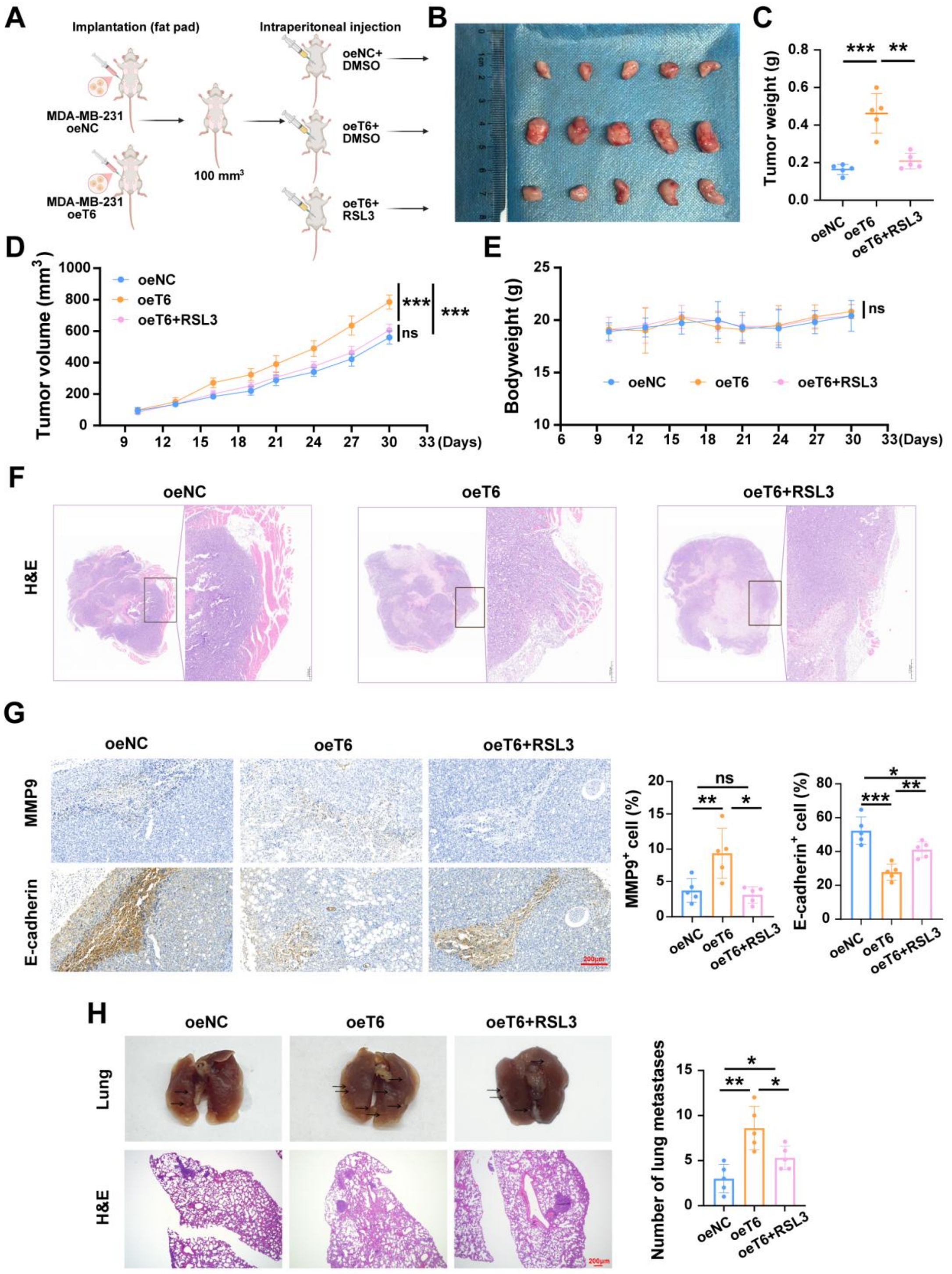

Supplementary Figure 8: RSL3 eliminates TRMT6 induced tumor migration and invasion *in vivo*.

(A) Schematic diagram to illustrate the treatment model protocol. **(B)** Representative images of tumors inorthotopic xenograft mice. **(C-E)** Statistical analysis of tumor

weight **(C)**, body weight **(D)**, and tumor volume **(E)** in different groups of nude mice (n = 5). Data were measured every three days. **(F)** Representative H&E images in serial segments of tumor tissue separated from the orthotopic model. Scale bar = 312.5 μm. **(G)** IHC images of MMP9 and E-cadherin expression and statistical charts from the orthotopic model. Scale bar = 200 μm. **(H)** Representative photographs and H&E staining of lung tissues from each treatment group. Scale bar = 200 μm. Significant differences were shown by $^{*}P < 0.05$, $^{**}P < 0.01$, and $^{***}P < 0.001$, ns, not significant.

Supplementary Table 1. Baseline information of patients with TNBC.

Characteristic	Number
**Age (mean ± SD)**	52.81 ± 7.5
**T stage, n (%)**	
T1	9 (33.33%)
T2	15 (55.56%)
T3	3 (11.11%)
T4	0 (0%)
**N stage, n (%)**	
N0	14 (51.85%)
N1	10 (37.04%)
N2	3 (11.11%)
N3	0 (0%)
**Pathologic stage, n (%)**	
Stage I	6 (22.22%)
Stage II	18 (66.67%)
Stage III	3 (11.11%)
Stage IV	0 (0%)

Supplementary Table 2. Sequences of primers used for RT-qPCR in this study.

Name	Sequence
TRMT6 Forward	GGTGCTGAAACGTGAAGATGT
TRMT6 Reverse	CTTGGGCTGTAGACTTCCTCC
WDR5 Forward	GGTGGGAAGTGGATTGTGTC
WDR5 Reverse	CAGCAGAGGCGATGATGTT
FTH1 Forward	TCCTACGTTTACCTGTCCATGT
FTH1 Reverse	GTTTGTGCAGTTCCAGTAGTGA
GAPDH Forward	CAGGAGGCATTGCTGATGAT
GAPDH Reverse	GAAGGCTGGGGCTCATTT
TRMT6 Promoter Forward	GCCAAGTCCGCAGTTCTCTTC
TRMT6 Promoter Reverse	TGAGTTTCCCGAGCCGTTGAG
RbBP5 Forward	TCTGCTCCTGTCATGTTGACC
RbBP5 Reverse	CTCGCCTATCAAAAGATGCCA
DPY30 Forward	GGAGGGACAAACGCAGGTT
DPY30 Reverse	GGTAGGCACGAGTTGGCAA
Ash2L Forward	ATGGCAGTCCCGAACACAG
Ash2L Reverse	GGTTGTCATGCACTCCCAGTAT

Supplementary Table 3. Sequences of siRNA and shRNA used in this study.

Gene name	Probe (5’-3’)
si-TRMT6#1	GGACAGTCTTCTACATGGA
si-TRMT6#2	GGTCTACTGTCAGTACAAA
si-WDR5	GCUCAGAGGAUAACCUUGU
si-FTH1	GAUGAAGCUGCAGAACCAA
si-RbBP5	GCTCTATTGTATTTACCCATT
si-DPY30	CTTGCTGTGCTTGCAAAGGAA
si-Ash2L	GCUGCACGGUUUCCAUUAATT

Supplementary Table 4. Primary antibodies used in this study.

Antibodies	Source	Identifier
Anti-TRMT6	Proteintech	Cat#16727-1-AP
Anti-WDR5	Zenbio	Cat#382160
Anti-FTH1	Proteintech	Cat#11682-1-AP
Anti-FTL	Zenbio	Cat#R40105
Anti-4HNE	Bioss	Cat#bs-6313R
Anti-GAPDH	Servicebio	Cat#ZB15004-HRP-100
Anti-Ki67	Zenbio	Cat#R381101
Anti-puromycin	Kerafast	Cat#3RH11
Anti-H3K4me3	Affinity	Cat#DF6935
Anti-m^1A	Abcam	Cat#ab208196
Anti-MYC tag	Proteintech	Cat#60003-2-1g
Anti-MMP9	Proteintech	Cat#10375-2-AP
Anti-E-cadreine	Proteintech	Cat#20874-1-AP
Anti-RbBP5	Affinity	Cat#DF4423
Anti-Ash2L	HUABIO	Cat#ET1609-24
Anti-DPY30	HUABIO	Cat#M1311-6
Anti-EpCAM	Affinity	Cat#DF6311
Anti-CD45	Zenbio	Cat#R380923
